# Diagnostic and therapeutic potential of exosomal circRNAs in cancer: decoding the circular code toward precision medicine

**DOI:** 10.1186/s12935-025-04053-w

**Published:** 2025-11-27

**Authors:** Nadia M. Hamdy, Mohamed H. Noureldein, Shaimaa A. Gouhar, Roba M. Talaat, Amira Mohamed Abd El-Jawad, Hekmat M. El Magdoub, Sherien M. El-Daly

**Affiliations:** 1 Biochemistry and Molecular Biology Department, Faculty of Pharmacy, Ain Shams University, Abassia, Cairo, 11566 Egypt; 2 Department of Neurology, Michigan Medicine, Ann Arbor, MI USA; 3 NeuroNetwork for Emerging Therapies, University of Michigan, Ann Arbor, MI USA; 4 Medical Biochemistry Department, Medical Research and Clinical Studies Institute, National Research Centre, Giza, Egypt; 5 Molecular Biology Department, Genetic Engineering and Biotechnology Research Institute (GEBRI), University of Sadat City, El Sadat, Egypt; 6 Biochemsitry Department, Faculty of Pharmacy, Misr International University, Cairo, Egypt; 7 Cancer Biology and Genetics Laboratory, Centre of Excellence for Advanced Sciences, National Research Centre, Giza, Egypt

**Keywords:** Exosomes, CircRNAs, Cancer biomarkers, Liquid biopsy, Non-coding RNAs, Precision medicine

## Abstract

Circular RNAs (circRNAs), a unique class of non-coding RNAs, have gained attention due to their stable closed-loop structure and involvement in diverse cellular processes. Their remarkable stability and abundance in biological fluids, particularly when encapsulated within exosomes, make them highly promising candidates for clinical applications. This review offers a detailed insight into the growing field of exosomal circRNAs, starting with the fundamental principles of circRNA biogenesis and the selective mechanisms governing their sorting into exosomes. We explore the complex regulatory network controlled by circRNAs, highlighting their roles as microRNA (miRNA) sponges, interactors with RNA-binding proteins (RBPs), regulators of transcription and RNA stability, and even as templates for translating into novel peptides or proteins. We elucidate how these actions are mediated by exosomal circRNAs when delivered to recipient cells, influencing the behavior of target cells. The review critically evaluates the current and emerging methodologies for the detection, quantification, and functional analysis of exosomal circRNAs. We extensively discuss the clinical implications, highlighting the significant potential of exosomal circRNAs as non-invasive biomarkers for the diagnosis, prognosis, and monitoring of various diseases, with a particular focus on cancer. Furthermore, we explore their therapeutic landscape, including strategies for targeted delivery, their role in modulating drug resistance, and their intricate functional crosstalk with other non-coding RNAs in cancer progression. While recognizing the promising potential of exosomal circRNAs, we also address the existing technical challenges and highlight the future directions needed to leverage their diagnostic and therapeutic applications. Our review highlights the significance of interpreting the circular code of exosomal circRNAs as a vital step toward achieving the objectives of precision medicine.

**Graphical Abstract:**

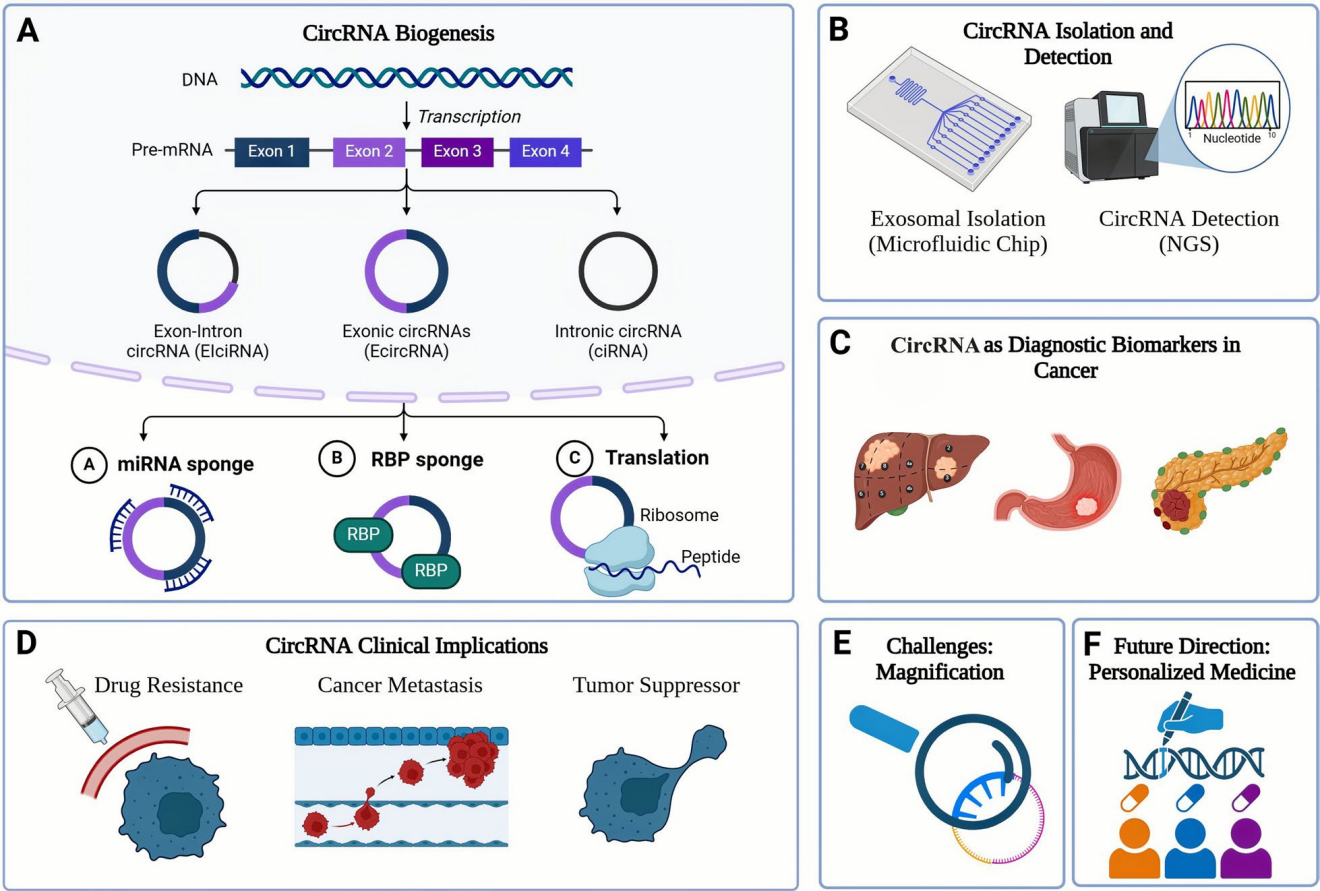

## Highlights

o Exosomal circRNAs represent a promising class of circulating biomarkers for early cancer detection, prognostic assessment, and monitoring treatment response.
o Exosomal circRNAs act as mediators in cell-to-cell communication, driving cancer progression or suppression through mechanisms like miRNA sponging and protein interaction.
o Certain exosomal circRNAs are directly implicated in the development of therapeutic resistance, offering a new axis for targeted interventions to enhance treatment efficacy.
o Standardization of detection methodologies and a deeper understanding of circRNA sorting mechanisms are critical next steps for translating exosomal circRNAs into clinical practice.

## Introduction

### Background

The human transcriptome contains a significant amount of RNA transcripts that are not translated into proteins, known as non-coding RNAs (ncRNAs) [1]. Those ncRNAs were initially thought of as non-functional or “junk DNA” sequences [2]. Research has shown that the proportion of ncRNA sequences increases with the complexity of an organism [2–4]. This suggests that ncRNAs, along with their irregular expression, could play a significant role in the development of human diseases. Additionally, they may provide valuable insights into the underlying mechanisms of various disorders [5]. This is primarily because ncRNAs influence complex cellular and molecular processes [6, 7].

According to their function, ncRNAs are categorized into structural (housekeeping) and regulatory RNAs. Structural ncRNAs are essential for cellular machinery and include transfer (tRNA), ribosomal (rRNA), small nuclear (snRNA), and small nucleolar (snoRNA) RNAs [8]. Regulatory ncRNAs that control gene expression are categorized into two main classes according to their length: small non-coding RNAs of less than 200 nucleotides, which include microRNAs[9, 10] (miRNAs), small interfering RNAs (siRNAs), long non-coding (lncRNAs) of more than 200 nucleotides [11–13] and PIWI-interacting RNAs (piRNAs) [14]. Additionally, other important classes exist, such as enhancer RNAs (eRNAs), transcribed from enhancers with variable lengths and circular RNAs (circRNAs), characterized by their covalently closed structure and varying lengths [15] both contributing significantly to cellular regulation beyond the primary length-based classification [16].

**Challenges:** Although circRNAs were first detected by electron microscopy about 50 years ago [17, 18], researchers initially paid little attention to their fucntion until a relevant number of studies revealed their resistance to degradation by exonucleases compared to linear RNAs. This stability was due to their special circular shape [18] with no 5′ caps or 3′ poly (A) tails [19, 20]. Since then, circRNAs have become a research focus.

A significant breakthrough in the study of circRNAs was the discovery of these molecules within exosomes. Exosomes are tiny vesicles secreted by various cell types that serve as vehicles for intercellular communication. The incorporation of circRNAs into exosomes not only protects them in the external environment but also aids in their transport to recipient cells, potentially influencing downstream signaling pathways [21, 22].

Exosomes were first identified in 1983 [23]. They are secreted by different types of cells into interstitial and body fluids, including cerebrospinal fluid (CSF), blood, saliva, urine, and breast milk [24, 25]. They are defined as nanoscale particles with phospholipid double-membrane-bound vesicles [26]. The exosomal biogenesis takes place via the inward budding of the plasma membrane and the development of multivesicular bodies (MVBs) [27].

Exosomal diameter ranges from 30 to 100 nm [28], and they are characterized by several distinct surface molecular markers, such as CD9, CD63, and CD81 [29, 30, 31].

Exosomes are released by nearly all cell types and serve as key mediators of intercellular communication, as they can transfer diverse cargo—including lipids, proteins, DNA, mRNAs, lncRNAs, and circRNAs—from donor to recipient cells. Through this cargo delivery, they modulate various aspects of cellular biology [32–34].

Exosomes have addressed many of the limitations associated with traditional biomarkers due to their stability, resistance to degradation, and ability to accurately reflect the molecular characteristics of parental cells. In contrast, protein-based biomarkers often struggle with sensitivity and specificity because their levels can be influenced by a variety of non-malignant conditions [35]. Circulating tumor DNA (ctDNA) and cell-free RNAs, while promising, are highly susceptible to enzymatic degradation and require advanced detection technologies that limit routine clinical use [36]. Similarly, traditional non-coding RNAs such as miRNAs and lncRNAs are unstable in circulation and show inconsistent reproducibility across studies [37]. In contrast, exosomal circRNAs combine the intrinsic stability of their covalently closed loop structure with the additional protection provided by the exosomal membrane, ensuring durability in biological fluids. Their tumor-derived origin confers high specificity, making them promising candidates to address the shortcomings of current biomarkers and to serve as robust tools for diagnosis, prognosis, and therapeutic monitoring.

**Rationale:** Over the past few decades, numerous studies on exosomal miRNAs and exosomal lncRNAs have been carried out [38, 39]. However, exosomal circRNAs have received comparatively little attention.

**Aim and objectives:** Therefore, this review aims to provide a comprehensive overview of exosomal circRNAs, emphasizing their unique biological features and multifaceted roles in cancer. We highlight their contribution to cellular communication, tumor progression, and therapeutic resistance, while also examining their potential as stable and specific biomarkers for diagnosis and prognosis. In addition, we will discuss advances in detection technologies, bioinformatic resources, and potential therapeutic applications of exosomal circRNAs, alongside the challenges that currently hinder their clinical translation. By integrating recent discoveries with ongoing debates and research gaps, this review seeks to clarify the promise and limitations of exosomal circRNAs and to outline future directions for their application in precision medicine.

### Biogenesis of circRNAs

CircRNAs comprise a unique class of non-coding RNAs that are widely expressed in cells [40]. Unlike the linear RNA transcripts that are generated by a canonical splicing process. CircRNAs are generated from pre-mRNA by back-splicing (tail-to-head circularization) [41]. Back-splicing takes place when both ends of a single pre-mRNA molecule connect covalently to form a closed circle, joining the 3’ splice site to the 5’ splice site, forming a "head-to-tail" circular molecule [42]. Based on their genomic origin and the specific sequences incorporated into the final circular structure, circRNAs can be categorized into three distinct classes of circRNAs: exon circRNAs (EciRNAs), exon–intron (EIciRNAs), and intron circRNAs (ciRNAs) [43, 44].

**Fig. 1:**
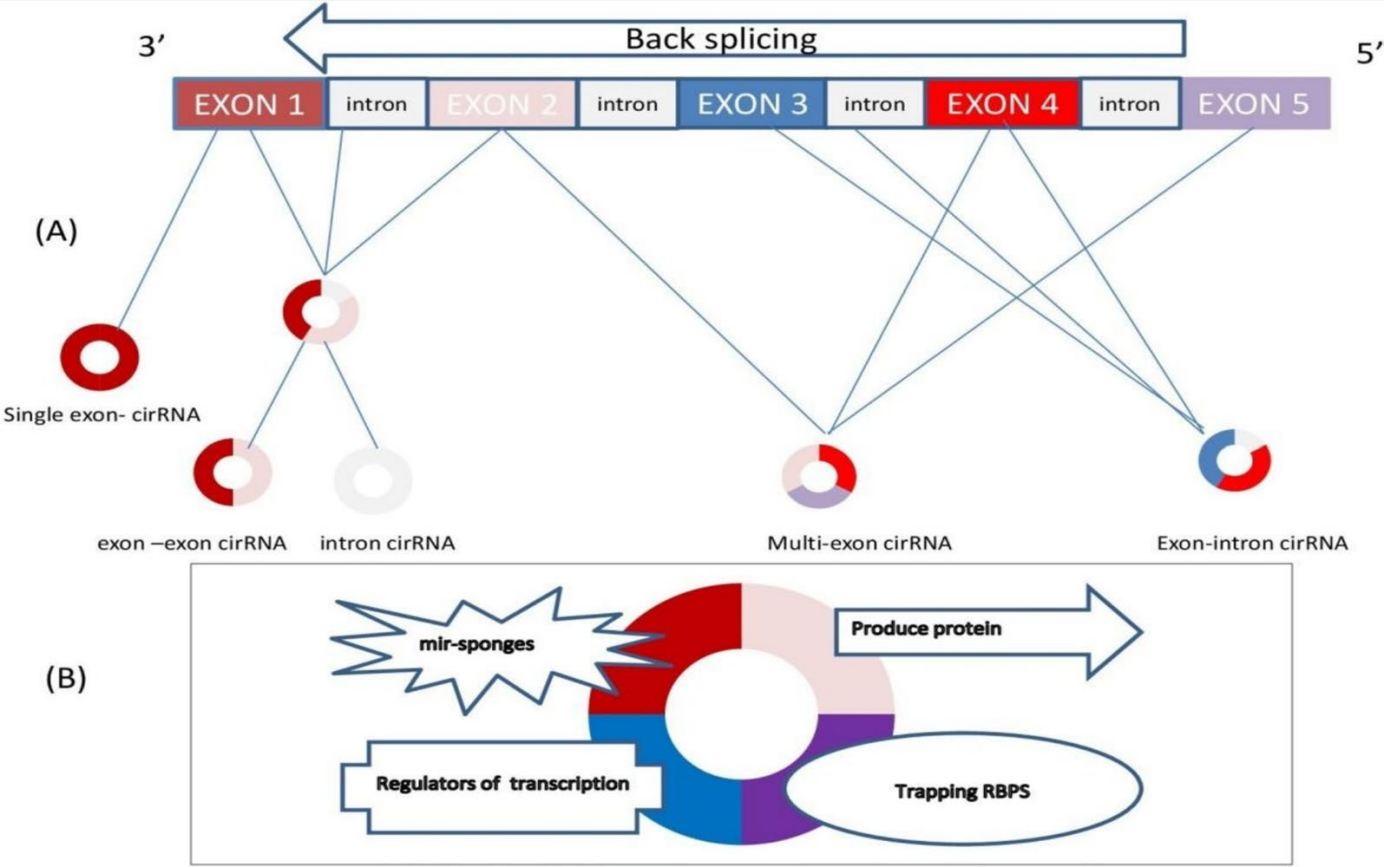
CircRNA biogenesis (**A**) and functions (**B**)

The exon circRNAs (EciRNAs) are formed by pre-mRNA splicing when the 3′ splice donor covalently links to the 5′ splice acceptor. This could involve either single or multiple exons. On the other hand, if the intron connecting the exons is maintained, an exon–intron (EIciRNA) structure forms, containing both exonic and intronic sequences due to back-splicing occurring before the removal of the intron is complete [45]. Finally, intron circRNAs (ciRNAs) are formed solely from introns that escape the standard debranching process [43] as shown in Fig. 1.

Recent studies have highlighted that circRNA biogenesis is not merely a stochastic splicing event but is tightly regulated by RNA-binding proteins (RBPs), complementary intronic sequences, and epigenetic modifications [46, 47]. Moreover, growing evidence indicates that a subset of circRNAs is selectively packaged into exosomes via active sorting mechanisms involving RBPs and specific sequence motifs. This selective enrichment in exosomes distinguishes exosomal circRNAs from their intracellular counterparts and confers enhanced stability, systemic bioavailability, and functional relevance in intercellular communication. It is worth noting that most cellular RNAs exist as ribonucleoprotein (RNP) complexes, and RNA-binding proteins (RBPs) play a critical role in mediating their selective loading into exosomes [48, 49]. Specific sequence motifs within the RNA can guide their transport to subcellular compartments, including exosomes, thereby enabling targeted extracellular communication [50]. For instance, the heterogeneous nuclear RNP A2B1 (hnRNPA2B1) has been shown to recognize GGAG motifs in various ncRNAs, including miRNAs, lncRNAs, and potentially circRNAs, facilitating their sorting into exosomes [51]. Functional studies in cancer models have demonstrated that disrupting hnRNPA2B1 or altering its motif recognition can selectively modify the exosomal content, affecting drug resistance and intercellular signaling [52–54]. These examples demonstrate that the selective packaging of RNAs—including circRNAs—into exosomes is a tightly regulated process orchestrated by RNA-binding proteins (RBPs) and specific sequence motifs. This motif- and RBP-dependent sorting not only ensures precise loading of circRNAs into exosomes but also confers them with enhanced stability, protection from degradation, and the ability to act systemically in recipient cells. Consequently, exosomal circRNAs represent a distinct subclass of circRNAs, functionally and biologically differentiated from their intracellular counterparts, with significant implications for intercellular communication, disease progression, and potential therapeutic applications.

While the fundamental mechanisms of circRNA biogenesis—back-splicing, RBP-mediated regulation, and complementary intronic pairing—are well established, studies on their selective enrichment into exosomes remain relatively preliminary. Most current evidence relies on in vitro cell line systems or proteomic analyses implicating RBPs such as hnRNPA2B1, rather than large-scale functional validations. This underscores that, although circRNA sorting into exosomes is clearly a regulated process, the specific determinants and their physiological relevance require further clarification in patient-derived or in vivo contexts.

The figure highlights the structural diversity and functional roles of circRNAs in cellular regulation. **(A)** circRNA Biogenesis: circRNAs are formed through back-splicing, generating different types: single-exon circRNA, exon-exon circRNA, intron circRNA, multi-exon circRNA, and exon–intron circRNA. **(B)** circRNA Functions: circRNAs regulate gene expression by acting as miRNA sponges, trapping RNA-binding proteins (RBPs), modulating transcription, and, in some cases, translating into proteins.

### Sorting of circRNA into exosomes

The relationship between the expression level of a circRNA and a mature mRNA originating from the same pre-mRNA is not always predictable and is not always correlated [55]. It has been observed that circRNAs are more loaded into exosomes than linear RNAs and that they are also more abundant in exosomes than in their producer cells [56, 57]. This variation could be attributed to different reasons. Primarily, circRNAs have a relatively long half‑life due to their covalently closed loop structure without 5’ or 3’ ends or poly-A tails making them more stable against exonucleolytic degradation. Thus, their transcripts can accumulate at higher levels compared with their linear counterparts [56]. Secondly, the release of circRNAs from cells in extracellular vesicles (EVs), as a clearance mechanism, may also explain their high abundance in exosomes [58]. Another possible mechanism involves exosomes themselves; being nanosized particles with a double‑layered membrane, they inherently prolong the circulation half‑life of circRNAs and enhance their biological activities [59].

## Mechanisms of circRNA action: A multifaceted regulatory network

**Fig. 2:**
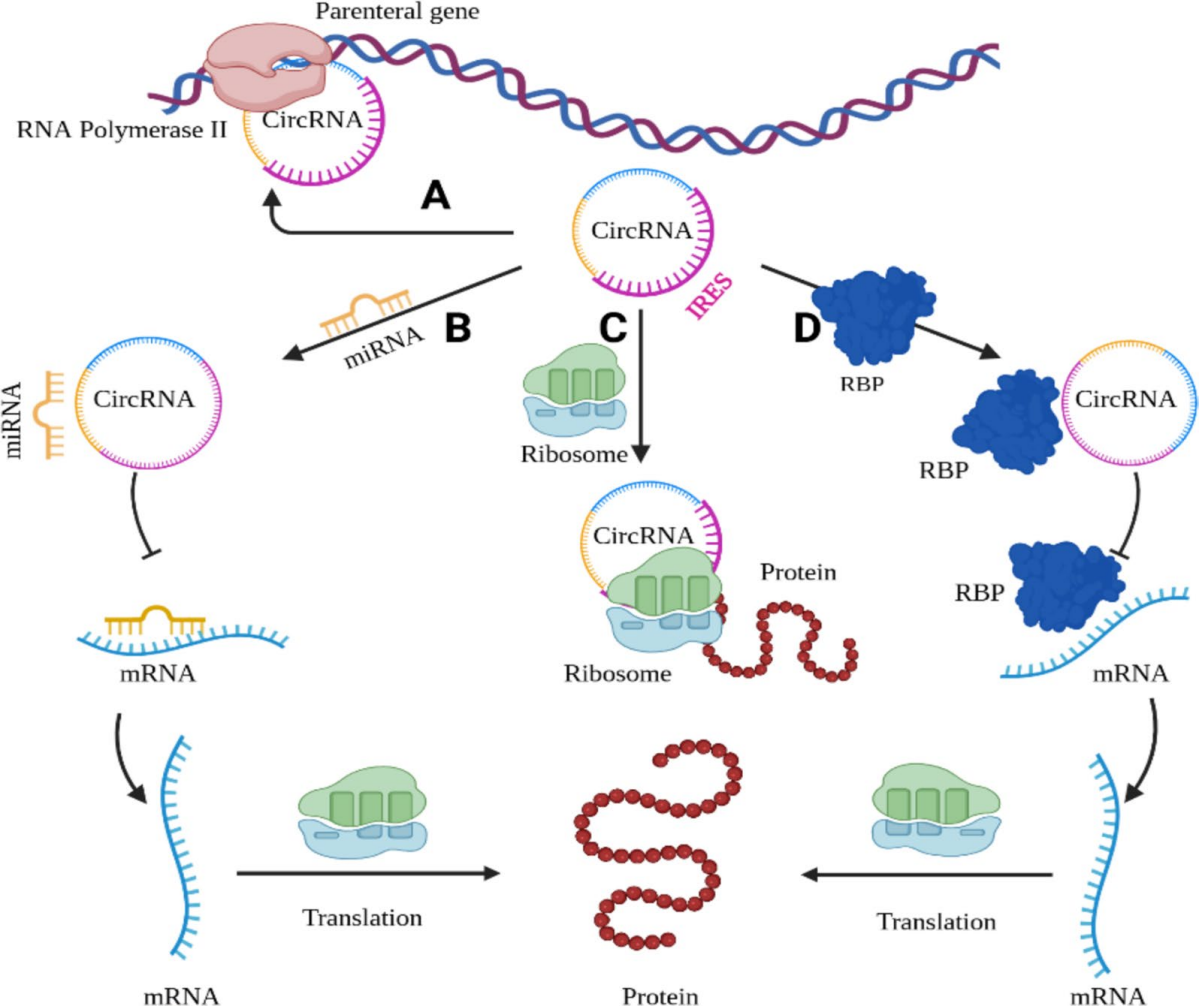
Regulatory mechanisms of circRNAs

CircRNAs regulate physiological processes through several mechanisms and molecular pathways, operating at multiple levels of regulation as shown in Fig. 2. The key modes of action include;

**MicroRNA (miRNA) sponging:** Many circRNAs act as miRNA sponges or competing endogenous RNAs (ceRNAs). circRNAs can absorb and isolate miRNAs, thus affecting their downstream target gene expression and controlling their action [60]. miRNAs usually target the 3′ untranslated regions (UTRs) of specific mRNA targets and regulate their stability and/or translation [61]. The level of complementarity between mRNA and miRNA determines the mechanism of miRNA action on target mRNA. As each miRNA can have complementarity with many mRNAs, they have the potential to regulate multiple genes [62]. miRNAs are shown to be involved in posttranscriptional regulation of gene expression in nearly all cellular events. Therefore, accumulating evidence indicates that circRNAs play a crucial role in gene expression regulation partly by inhibiting miRNA activity. They competitively bind to miRNAs as ceRNAs, known as miRNA sponges [63]. Many circRNAs act as competitive endogenous RNAs (ceRNAs) by sequestering miRNAs and preventing them from binding to their mRNA targets. For example, circUHRF1 sponges miR-526b-5p, leading to c-Myc activation, promoting HCC cell proliferation and invasion [64]. Also, circHIPK3: Acts as a “super sponge,” binding multiple miRNAs such as miR-124, thereby facilitating cancer cell growth and survival [65]. In CRC, circ_0005963 enhances oxaliplatin resistance by sponging miR-122, which upregulates PKM2, promoting glycolysis [66].

**Interaction with RNA-binding proteins (RBPs):** circRNAs can seize RBPs trapping them in the cytoplasm, hence, preventing their nuclear entry and regulating their function [67]**.** CircRNAs can bind RBPs, altering their localization or function, which significantly impacts gene regulation and cancer progression. For example, circRNA_100338 interacts with NOVA2, a splicing regulator, influencing RNA processing and metastasis in HCC [68]. These interactions enable circRNAs to act as scaffolds, recruiting RBPs to specific transcripts or preventing their nuclear entry, thus shaping cancer cell behavior.

**Regulation of transcription:** Certain circRNAs, especially those retained in the nucleus (e.g., EIciRNAs), can bind with some transcriptional factors in the nucleus, forming a complex that interacts with the RNA polymerase II machinery, regulating their parental genes’ transcription [69, 70]. For example, circEIF3J and circPAIP2 form complexes with U1 snRNP and RNA polymerase II, enhancing the transcription of their source genes [19]. This mechanism underscores the dual role of circRNAs as both post-transcriptional regulators and nuclear modulators of gene expression.

**Modulation of RNA stability:** Beyond indirectly affecting mRNA stability via miRNA sponging, some circRNAs can directly influence the stability of other RNA molecules. This can occur through direct base-pairing interactions with target RNAs or by recruiting specific proteins that either stabilize or destabilize associated transcripts [45, 71].

**Translation into peptides/proteins:** Although primarily considered non-coding, several studies reported that a subset of circRNAs can be translated into functional peptides or proteins [72] and the dysregulation of these proteins could lead to cancer or other diseases [73]. Some circRNAs contain internal ribosome entry sites (IRES) and open reading frames (ORFs), allowing translation into small peptides that influence tumor progression [74]. In case of CRC, circRNA-ATG4B encodes a 222-aa protein that enhances autophagy and chemoresistance to oxaliplatin [75]. This emerging role adds complexity to circRNA biology and introduces potential targets for therapeutic intervention.

While the canonical mechanisms of circRNAs—such as miRNA sponging, RBP interactions, transcriptional regulation, and translation—are well established, their encapsulation within exosomes adds a distinct functional dimension. For instance, exosomal circRNAs can be transferred to recipient cells where they continue to act as miRNA sponges and modulate signaling pathways that affect proliferation, drug resistance, and microenvironment interactions [76, 77]. This vesicle-mediated delivery enhances the stability and bioavailability of circRNAs, allowing them to participate in systemic intercellular communication and drive malignant behavior [78]. Furthermore, the translational potential of exosomal circRNAs enables the production of functional peptides or proteins in recipient cells, thereby adding an extra layer of regulatory influence absent in conventional intracellular circRNAs [79]. Collectively, these findings underscore that exosomal circRNAs are not merely intracellular regulators but dynamic mediators of systemic cellular crosstalk, with unique implications for cancer biology and therapeutic targeting.

Collectively, these mechanisms—miRNA sponging, RBP binding, transcriptional regulation, and translation—form the foundation of circRNA biology. However, much of the mechanistic work remains descriptive and is frequently confined to cell-based assays. The translation of circRNAs into peptides, while conceptually exciting, is supported by limited experimental evidence, often restricted to ribosome profiling or small-scale validations. This highlights the need for caution when extrapolating mechanistic models to clinical contexts, emphasizing that exosomal circRNAs require more robust, functional demonstration of these activities.

Figure 2 illustrates the primary mechanisms by which circRNAs regulate gene expression and protein synthesis **(A)** forming a complex to regulate their parental genes’ transcription, **(B)** sponging miRNAs, **(C)** direct translation with ribosomal assistance when containing an IRES, and **(D)** interacting with RBPs. [RBP: RNA-binding protein, IRES: Internal ribosome entry site.]

## Decoding the action of exosomal circRNAs: sponging, binding, and translation

Li et al. (2015) discovered that circRNAs were surprisingly plentiful, persistent, and able to move across cells through exosomes. Their results indicated that exosomes contained at least twice as many circRNAs as the circRNA-producing cells themselves [80].

As previously stated, the most widely studied function of circRNAs is acting as miRNA sponges, thereby eliminating the inhibition of miRNA on target genes and hence, increasing the latter expression levels [81]. The transfer of exosomes makes the circRNAs within them more dynamic, allowing them to regulate more downstream target genes. Two scenarios could arise: circRNAs first attach to miRNAs and enter exosomes together. When miRNAs reach the recipient cells, they separate from circRNAs and attach to mRNAs there, silencing them [82]. This illustrates how circRNAs bind to the corresponding miRNAs through complementary sequences, thereby controlling the expression of downstream target genes inhibited by miRNAs. A second hypothesis is that circRNAs reduce the inhibitory effect of miRNAs on the corresponding mRNA by absorbing the former in recipient cells after entering through exosomes [83].

CircRNAs have miRNA response element (MRE) sequences that enable them to bind to miRNAs. Because of this interaction, miRNAs are unable to limit the translation of mRNA into proteins. CircRNAs can therefore change the epigenetic regulation of gene expression or regulate the amount of the protein encoded by a particular gene. The interaction between miRNAs and circRNAs is especially significant in cancer development because it can either suppress or promote carcinogenesis, depending on whether tumor suppressor genes or oncogenes are present downstream of the target miRNA [84, 85]. One study was carried out on a ciRS-7, a special type of circRNA sponge for microRNA-7 (miR-7). ciRS-7, also known as CDR1as, can influence the binding capacity of miR-7 with target mRNAs. This study further suggests a competition between ciRS-7 and mRNAs for miR-7 binding [86].

A single circRNA can bind to multiple miRNAs at several sites. One example is circFOXO3, which can bind different miRNAs such as miR-138, miR-22, miR-762, miR-136, miR-433, miR-3622b-5p, miR-149, and miR-3614-5p. Another example is circITCH, which can bind to miR-7, miRNA-17, and miR-214 [87, 88].

CircRNAs exhibit a greater preferential binding to miRNAs than other endogenous RNAs (e.g., lncRNAs), giving them the name “super sponge” [89]. Xie et al. (2020) [90] found that exosomal circSHKBP1 increased HUR expression in gastric cancer (GC) tissues by sponging miR-582-3p. HUR is a part of the vascular endothelial growth factor (VEGF) signaling pathway that promotes VEGF secretion and induces angiogenesis, thereby promoting GC metastasis. In triple-negative breast cancer (TNBC), a previous study demonstrated that exosomal circPSMA1 promotes breast cancer (BC) cell metastasis by sponging miR-637, leading to the activation of the AKT1/β-catenin signaling pathway, which regulates cell proliferation and migration. High expression of AKT1 and low expression of miR-637 are highly correlated with poor prognosis in TNBC patients with lymph node metastasis [91]. CircPSMA1 overexpression has also been shown to significantly enhance the metastatic capacity in the liver and lungs of mice [92]. Zeng et al. (2020) found that overexpression of circFNDC3B severely inhibits angiogenesis in a mouse model of colorectal cancer (CRC). Exosomal circFNDC3B treatment inhibits CRC cell growth, angiogenesis, and liver metastasis in vivo [93].

CDR1as, an abundant circRNA in the mammalian brain, contains more than 70 conserved miR-7 and miR-671 binding sites that are completely complementary to the entire mature miR-671 sequence and can directly regulate the function of miRNAs and their collaborative gene [94]. CircHIPK3 has been shown to promote cancer cell growth by binding to multiple miRNAs, including the tumor suppressor miR-124 [65]. The existing results significantly demonstrate that circRNA-miRNA interaction is critical for the organism.

CircRNAs derived from introns cannot act as sponges to absorb miRNAs due to their limited number of binding sites. However, these circRNAs can enhance the expression of parental genes by regulating RNA polymerase II (RNA Pol II). Similarly, the interaction between EIciRNAs and U1 snRNP promotes the transcription of their parental genes in cis, highlighting a regulatory strategy for transcriptional control via specific RNA-RNA interactions [45, 95].

CircRNAs can also interact with proteins. CircDNMT1 promotes nuclear translation by interacting with P53 and AUF1, leading to autophagy or reduced instability of target mRNA [96]. In addition, Memczak et al. (2013) confirmed that circRNA-CDR1as can bind closely with AGO proteins, predominantly enhancing gene expression through miRNA regulation [82]. Further, circRNAs that contain an internal ribosomal entry site (IRES) can cause ribosomal recruitment and translation initiation, while circRNAs lacking IRES cannot encode proteins [97].

RNA-binding proteins (RBPs) form a large class of regulators that influence metabolism through their interactions with RNA. A recent study indicates that the human genome encodes more than 1500 RBPs [98]. A study by Zang et al. (2020) suggests that RBPs are involved in almost every aspect of the circRNA lifecycle, including generation, post-transcriptional regulation, functional execution, translation, and potential extracellular transport pathways. The authors emphasize the significant role of RBPs in circRNA maintenance and function [98]. In addition, exosomal circRNAs can interact with RBPs in recipient cells, potentially influencing biological processes. CircRNAs can function as dynamic scaffold molecules to competitively bind RBPs to their mRNAs. Huang et al. (2020) [68] have demonstrated that circRNA_100338 can bind to the RBP NOVA2, which is involved in the post-transcriptional regulation of RNA. In contrast to previous studies, Xu et al. (2020) provide substantial evidence that exosomal circRNAs can interact with binding proteins and regulate tumor growth in recipient cells. Once delivered, circRNAs bind to the RBPs in target cells, where they execute specific biological functions, such as influencing tumor growth [99]. This highlights the important role of exosomal circRNAs and their ability to regulate cellular activities through interactions with RBPs.

CircRNA–RBP interactions represent a critical but underexplored layer of cancer regulation. These complexes can stabilize oncogenic RNAs, modulate splicing, and control translation, directly impacting tumor growth, metastasis, and therapy resistance. Targeting such interactions may provide novel therapeutic opportunities, especially in cases where circRNAs and RBPs act synergistically to sustain malignancy. Taken together, these findings suggest that exosomal circRNAs can do canonical circRNA functions in recipient cells, extending their regulatory influence beyond the cell of origin. Nonetheless, most of the cited studies remain at the preclinical stage, relying on xenograft models and small patient cohorts. While these findings strongly implicate exosomal circRNAs in tumor growth, angiogenesis, and metastasis, the evidence base is still developing, and larger patient-derived datasets will be essential to confirm their clinical relevance.

## Integrative mechanistic model of exosomal circRNA function in cancer

Exosomal circRNAs act as central regulatory nodes in cancer biology, orchestrating multiple layers of gene expression control. When secreted into exosomes, these circRNAs travel through the tumor microenvironment and bloodstream, delivering their regulatory cargo to recipient cells. Their actions converge on three major oncogenic processes: drug resistance, immune evasion, and metastasis & angiogenesis. For example, circ_0005963 sponges miR-122, activating PKM2-mediated glycolysis, conferring oxaliplatin resistance in CRC [100]. While circRNA-ATG4B encodes a 222-aa peptide that enhances autophagy, contributing to CRC chemoresistance [75].

Regarding immune evasion, circUHRF1 (HCC) inhibits NK cell function by sponging miR-449c-5p, upregulating TIM-3, and promoting resistance to anti-PD-1 therapy [101]. Another example, circ_0001068 (OC) elevates PD-1 expression in T cells via miR-28-5p regulation [102]. The third major action of exsosomal circRNAs is metastasis & angiogenesis. In this context, circPSMA1 activates the AKT/β-catenin pathway via miR-637 sponging, promoting TNBC metastasis [91]. Also, circSHKBP1 induces GC angiogenesis through VEGF signaling [90]. The common pathways affected are PI3K/AKT, Wnt/β-catenin, and VEGF signaling in proliferation and metastasis, PD-1/PD-L1 axis in immune evasion, and autophagy and glycolysis pathways in drug resistance. These observations suggest a unifying model in which exosomal circRNAs serve as modular regulators, integrating post-transcriptional, protein-interaction, and translational mechanisms to shape cancer phenotypes. By coordinating multiple signaling cascades across cellular compartments, exosomal circRNAs drive the hallmarks of cancer, including sustained growth, metastasis, immune escape, and therapy resistance.

This schematic illustrates the central role of exosomal circular RNAs (circRNAs) as multifunctional regulators in tumor progression. Once packaged into exosomes and delivered to recipient cells, circRNAs modulate oncogenic signaling through three major mechanisms: **(1) miRNA sponging**, which releases oncogene expression and drives pathways such as PI3K/AKT and Wnt/β-catenin; **(2) RNA-binding protein (RBP) interactions**, influencing RNA stability, splicing, and protein localization; and **(3) translation into functional peptides**, which promote survival and metabolic adaptation. Collectively, these mechanisms converge to enable critical cancer phenotypes, including **drug resistance** (e.g., via glycolysis and autophagy regulation), **immune evasion** (e.g., through PD-1/PD-L1 axis modulation), and **metastasis and angiogenesis**. This integrated framework highlights exosomal circRNAs as nodal regulators within shared oncogenic networks, underscoring their potential as biomarkers and therapeutic targets in precision oncology.

## Detection and analysis methods for exosomal circRNAs

There is currently no universally accepted gold standard method for isolating and purifying exosomal circRNAs. The following section will discuss the most commonly used techniques for exosome isolation and circRNA extraction, as well as the challenges associated with these methods and recommendations for improvement.

### Techniques for exosomes isolation and purification

Isolating exosomes is challenging due to their small size (50–200 nm), heterogeneity, and fragility [103]. For clinical diagnostic purposes, exosomes must be separated from complex body fluids such as blood [104], pleural effusion [105], ascitic fluid [106], breast milk [107], saliva [108], and urine [109]. These fluids contain cells, proteins, and other materials that interfere with the isolation process, reducing the purity of the exosomes. Consequently, there is a need for simple and specific techniques for exosomes isolation and purification.

Currently, there are various techniques that can be employed, including ultracentrifugation, magnetic bead separation, gel exclusion, immunoaffinity capture, and lectin-induced exosome aggregation or precipitation [110]. Numerous commercial kits for exosome isolation, with various yield and purity specifications, are also available for use. Additionally, new isolation methods have been developed based on the granularity or other inherent characteristics of exosomes [111].

The most common exosome isolation technique is ultracentrifugation, which uses high centrifugal force (10,000 × g) to precipitate exosomes. Although straightforward, this method has several limitations, including the need for bulky and costly equipment, time-consuming processing, low exosome purity, and the requirement for large samples. To address these limitations, sucrose-gradient centrifugation was introduced, resulting in gradient ultracentrifugation. This technique uses a gradient density solution to separate vesicles of different sizes, achieving higher purity exosomes. However, it still requires bulky and expensive equipment, and large sample volumes [111].

Co-precipitation isolation kits, such as ExoQuick (System Biosciences, CA, USA) and Exo-Spin (Cell Guidance Systems LLC, MO, USA), have been developed to eliminate the need for bulky ultracentrifugation equipment. These kits use reagents to reduce exosome solubility, leading to their precipitation. The precipitated exosomes can then be isolated using low centrifugal force [112, 113]. However, the reagents in these kits also reduce the solubility of lipoprotein and Ago-2 RNA, compromising the purity of the isolated exosomes. Additionally, these kits are expensive which limits their use in large-scale experiments or routine clinical investigations [114].

The need to separate exosomes from complex body fluids with high content of contaminating proteins has led to the adoption of size exclusion gel chromatography for exosomal isolation. This technique uses a gel matrix to separate particles based on their size. It effectively isolates high-purity exosomes from complex biological fluids, such as blood and urine. Columns with small pore sizes (75 nm) are utilized to isolate exosomes larger than 75 nm, allowing them to pass through while trapping smaller protein complexes for later elution. Like other chromatography methods, efficient separation depends on several factors including column type, media, pore size, and flow rate [115–117].

Field flow fractionation is another interesting method that is based on laminar flow and Brownian motion physics. In this technique, a sample is introduced into a channel with a specific flow pattern that creates a laminar flow, and a perpendicular force field is applied to separate particles according to their size and molecular weight [111].

Additional techniques based on size separation are also being actively developed. One method uses microfluidic filtering to process biological fluids and collect exosomes in a collection channel. Although it can filter up to 300 µL-sample in less than 10 min, it is still considered to have low throughput [118]. Another technique, the deterministic lateral displacement (DLD) array, separates exosomes based on their size. In a specific DLD microfluidic chamber, larger exosomes follow the laminar flow, while smaller particles are displaced laterally across the array; however, this method has low throughput as well [119].

A contact-free sorting method developed by Lee et al. (2015) uses acoustic waves to fractionate exosomes. The radiation pressure from the acoustic waves causes particles to migrate to pressure nodes, with larger vesicles moving faster. This versatile system isolates pure exosomes [120].

Immunoaffinity capture is another effective approach for exosome isolation. Specific antibodies against known exosomal markers are used to precipitate exosomes, significantly enhancing isolation selectivity. A microfluidic device utilizing immunomagnetic beads has been developed for this purpose. This method is fast and straightforward but is marker-dependent and may underestimate exosome content. Additionally, it is challenging to separate the isolated exosomes from the magnetic beads for subsequent analysis [121].

These new microfluidic techniques are promising. However, further studies are needed to validate these techniques. Additionally, without cost-reduction and adaptation of these techniques for large scales, the use of these novel techniques would be limited.

### Emerging technologies for profiling and analyzing exosomal circRNA

Exosomes carry a variety of cargo, including proteins, DNA, and RNA, with RNA being the main nucleic acid component. The RNA found in exosomes consists primarily of noncoding RNA species or fragmented mRNAs [122, 123]. This noncoding RNA cargo includes miRNA and circRNA, which can be transferred to recipient cells and influence gene expression [124].

The most commonly employed methods for RNA extraction from exosomes involve phenol–chloroform extraction and spin column techniques. These methods, which are available in commercial kits such as Cytoplasmic and Nuclear RNA Purification Kit (Norgen, Ontario, Canada) and RNeasy kit (Qiagen, MD, USA), yield high-quality RNA. After extraction, the quality, size, and yield of the RNA are assessed using a Bioanalyzer System. The extracted RNA is then selectively amplified using a polymerase chain reaction (PCR) [125, 126].

To enrich for circRNA, we can add RNase R to degrade linear RNAs [127]. However, RNase R fails to digest > 20% of linear RNAs [127]. To increase the purity of circRNA, specific circRNA isolation kits have been developed with excellent specificity for circRNA such as abm’s circRNA Isolation Kit (Applied Biological Materials Inc, BC, Canada) which includes a poly(A)-polymerase to catalyze adenosine addition to the 3’ termini of all linear RNA which results in RNAs exhibiting a long, 3’ unstructured overhang. This overhang will make linear RNAs susceptible for degradation by RNase R, included in the kit, leaving highly pure circRNAs [127].

A modification for this method has been proposed, which removes linear RNA to near completion [128]. It involves RNase R digestion followed by adding poly(A)^+^ and poly(A)^+^ RNA Depletion (RPAD) [128, 129]. This method resulted in two interesting discoveries: **(1)** many EcircRNA isoforms share an identical back-splice sequence despite having different body sizes and sequences, and **(2)** thousands of novel IcircRNAs are expressed in cells [128].

Another method applies routine isolation of all RNA species using RNA 9000 Purity & Integrity kit (SCIEX, MA, USA) followed by using a pre-assembled, bare-fused silica multi-capillary cartridge and a high-throughput electrophoresis system (BioPhase 8800 system) to separate circRNA from mRNA. This method using a simplified sample preparation yielded high-resolution separation of circRNAs from linear RNAs as circRNAs exhibit delayed migration compared to the linear precursors. Interestingly, this method succeeded in detecting complex species, such as RNA concatemers, in addition to highly pure intact circRNA, by utilizing capillary gel electrophoresis with a laser-induced fluorescence detector [130].

To identify specific circRNA species in exosomes, either real-time PCR (RT-PCR) or conventional PCR followed by gel electrophoresis can be utilized. For high-throughput detection of all circRNAs and to identify novel circRNAs, next-generation sequencing (NGS) is employed. In addition to identifying circRNA, NGS can detect other coding and noncoding RNA species isolated from exosomes. NGS also has the capability to identify novel and previously uncharacterized RNA species [131, 132].

After reviewing methods for exosomal isolation, circRNA extraction and detection, we can conclude the following recommendations: (1) choice of the extraction method should be guided by the study objective. Specialized kits are recommended but the cost can be high for large scale studies; (2) New microfluidic techniques for exosome isolation can increase exosome purity but they are costly and not widely used or validated for large scale studies; (3) circRNA should be enriched during RNA isolation either using kits, preamplification, RNase R or combination of all of them; and (4) NGS sequencing is the method of choice for circRNA detection from exosomes.

### Computational approaches for circRNA identification and analysis

Successful exosome isolation and circRNA sequencing are only the first steps in circRNA identification. Sequencing is followed by rigorous computational analysis to mine the huge high throuhput data to detect circRNA. Only very few studies have compared the functionality and usability of different circRNA identification tools [133–136]. Table 1 lists computational tools used in circRNA identification. While a comprehensive assessment of all these tools is beyond the scope of this review, we will briefly discuss five of the most commonly used tools, highlighting their underlying algorithms, strengths and limitations as well as offering recent recommendations for future circRNA detection and validation.

**Table 1:** Lists of the common computational tools used in circRNA identification

Tool	Link
**Entire genome screening**	
CIRCexplorer	https://circexplorer2.readthedocs.io/en/latest/
Circseq_cup	https://github.com/bioinplant/circseq-cup
Circtools	https://docs.circ.tools/en/latest/
CIRI2	https://ciri-cookbook.readthedocs.io/en/latest/CIRI2.html
CIRIquant	https://github.com/bioinfo-biols/CIRIquant
find_circ	https://github.com/marvin-jens/find_circ
KNIFE	https://genboree.org/theCommons/projects/exrna-tools-may2014/wiki/KNIFE_(Known_and_Novel_IsoForm_Explorer)
PFv2	https://github.com/osagiei/pfv2
Sailfish_cir	https://sailfish-cir.readthedocs.io/en/stable/main.html
segemehl	http://legacy.bioinf.uni-leipzig.de/Software/segemehl/
CircMiner	https://github.com/vpc-ccg/circminer
FcircSEC	http://hpcc.siat.ac.cn/FcircSEC/Home.html
MapSplice	https://github.com/LiuBioinfo/MapSplice
Circall	https://github.com/datngu/Circall
circRNAFinder	https://github.com/bioxfu/circRNAFinder
CircRNA-Pro	https://hub.docker.com/repository/docker/songweidocker/circrna_pro
**Reference-free tools**	
CIRIT	https://www.bio-add.org/CIRIT/
**Plant-Specific**	
CircPlant	http://bis.zju.edu.cn/circplant
PCirc	https://github.com/Lilab-SNNU/Pcirc
**Known splice sites screening**	
CircSplice	https://gb.whu.edu.cn/CircSplice/userguide.html
NCLscan	https://github.com/TreesLab/NCLscan
**Specific for long reads**	
CIRI-long	https://github.com/bioinfo-biols/CIRI-long
IsoCIRC	https://github.com/Xinglab/isoCirc
circNICK-Irs	https://github.com/omiics-dk/long_read_circRNA
**Frameworks**	
circRNAprofiler	https://www.bioconductor.org/packages/devel/bioc/vignettes/circRNAprofiler/inst/doc/circRNAprofiler.html
circRNA_finder	https://github.com/orzechoj/circRNA_finder
UROBOROS	https://github.com/WGLab/UROBORUS
**Integrative tools**	
CirComPara2	https://github.com/egaffo/circompara2
NCLcomparator	https://github.com/TreesLab/NCLcomparator
**Databases**	
circBase	https://www.circbase.org
TSCD	https://ngdc.cncb.ac.cn/databasecommons/database/id/3675
ExoRBase	http://www.exorbase.org

#### General workflow

A general workflow pipeline of circRNA identification includes alignment, scanning alignment files to detect back splicing events, filtration to remove false positives, quantification and annotation. Various aligners can be used to align sequencing reads against a reference genome such as Bowtie2, BWA-MEM, HISAT2, or STAR. Most tools were validated using a specific aligner mentioned in their documentations. However, other aligners can be used with the same tool according to user’s preference. The second step in the identification is scanning the alignment files to detect back-spliced events. Computational tools differ in their stringency in this step. Some tools use back-spliced junctions, paired chiastic clipping or detection of chimerically aligned reads. Some tools apply a filtration step by checking for paired-end mapping and specific splicing signals. Finally, some integrated tools and workflows can perform quantification and annotation of known circRNAs through a database connection.

#### CircRNA identifier (CIRI)

CIRI is one of the most widely used tools for circRNA identification from RNA-seq data [137]. It is a robust method that applies several scanning and filtration steps to ensure correct unbiased circRNA detection. After aligning the sequencing reads using an aligner such as BWA-MEM, the CIRI algorithm starts by scanning the SAM alignment files twice to detect back-spliced junction (BSJ) reads and paired chiastic clipping (PCC) signals reflecting circRNA candidates. Both BSJ and PCC are strong markers for circRNAs. BSJ is detected when a single read maps to two distinct, non-consecutive sites om a reversed orientation in the genome while PCC signal is detected when the read’s segments are clipped and mapped to different genomic locations in a chiastic or cross-shaped pattern. circRNA candidates are then scanned for primary filtration to remove false positives through checking for the presence or paired-end mapping (PEM) and GT-AG splicing signals. A second filtration step is applied to find additional junction reads and apply more stringent filters. This step would remove false positives attributed to homologous genes and repetitive sequences. Finally, an output file will be generated with the list of identified circRNAs [137].

Since its development in 2015, CIRI has been continuously updated, maintained and improved. The CIRI suite is considered the most comprehensive circRNA identifier. The suite currently includes (1) CIRI2, which is an improved vesion of the original algorithm with options for multiple seed matching and multithreading [138]; (2) CIRI-full, which reconstructs the full-length sequence of circRNA isoforms; (3) CIRI-long, which was developed to identify circRNAs from long-read sequencing data such as Oxford Nanopore technology [139]; (4) CIRI-AS, which detects internal components and alternative splicing events within circRNAs; (5) CIRI-vis, which visualized outputs from CIRI; (6) CIRI-deep, which profiles circRNAs from single-cell and spatial transcriptomics data [140]; and (7) circAtlas v2.0, which is a comprehensive database of more than one million circRNAs across 6 species and different tissues. A CIRI-cookbook is available at https://ciri-cookbook.readthedocs.io/en/latest/CIRI2.html. CIRI has been successfully used to identify circRNAs in human [141], zebrafish [142, 143] and plants [144, 145].

#### Find_circ

Find_circ is a computational tool written in Python to detect head-to-tail spliced sequencing reads which reflect circRNA candidates. It was first published in the analysis of Memczak et al. in 2013 [82]. Similar to CIRI, it first starts by mapping the sequencing reads using Bowtie2 followed by discarding linear splice junctions and identifying chimeric reads which reflect circRNA back-splicing. Find-circ also includes a filtration step to remove linear splice junctions and retain only circular ones.

find_circ has many strengths including relying on a de novo approach in circRNA identification, easy command-line interface and integration into larger pipelines such as CircRNAwrap, and inclusion of filtration options. However, its performance is sensitive to read lengths with better sensitivity achieved with longer reads. find_circ is hosted on github with examples and tutorials (https://github.com/marvin-jens/find_circ).

#### CIRCexplorer

CIRCexplorer and its successor CIRCexplorer2 are modular pipelines for circRNA identification [146]. It is an integrated tool with modules for alignment, parsing, annotation and de novo assembly. This modular structure allows extensive flexibility for example various aligners can be used and integrated into CIRCexplorer. The use of the splice-aware aligner, HISAT2 with CIRCexplorer can enhance its ability to detect circRNAs. It uses an algorithm analogus to CIRI where circRNAs are identified through BSJs. An updated version called CIRCexplorer3-CLEAR was specifically designed to address the challenges of comparing circRNA and linear RNA expression by normalizing both to the same units [147]. CIRCexplorer is hosted on github at https://github.com/YangLab/CIRCexplorer2.

#### Detection of circular RNAs (DCC)

DCC is a program written on python for the detection and quantification of circRNA with high specificity. It takes the output from STAR aligner and identify circRNAs from chimerically aligned reads which contains circRNA junction spanning reads. DCC requires the specification of a repetitive region file in GTF format for filtering. This step makes using DCC more user-involving than other tools. DCC flexibility is extended through the use of many flags that facilitate annotation, filtering out candidates from mitochondrial genes, and removing candidates that span more than one gene. DCC output is comprehensive, and the output folder includes 4 files: CircRNACount, CircCoordinates, LinearCount and CircSkipJunctions.

#### Circminer

CircMiner is a recent tool for circRNA identification [148]. It adopts a pseudo-alignment approach to rapidly align and identify circRNAs. Since alignment is the most computationally and time intensive step, circMiner has superior accuracy and speed compared to other circRNA detection tools. It is available at https://github.com/vpc-ccg/circminer.

#### Challenges and recommendation for circRNA experiment design and analysis

The field of circRNA research is still in its infancy. There are no standard guidelines or benchmarked computational tools available for circRNA identification. Moreover, the results of circRNA identification tools may exhibit false positives due to back-splicing artifacts. Multiple measures can minimize the impact of these limitations. We have compiled some recommendations and present them as follows: (1) During RNA isolation, a selective method for circRNA enrichment should be used. Some of these methods are presented in the previous section. This would decrease impurities and false positives detection; (2) the use of long read sequencing techniques can improve the efficiency of circRNA detection, (3) circRNA should be identified based on analysis using 2 or more computational tools with high precision. Bioinformaticians should be aware of the tool’s detection approach and circRNA biology. Some integrative tools are available that combine the results of multiple circRNA detection tools, such as cirComPara2 and ecircscreen; (4) filtration of circRNAs based on minimum BSJ count; (5) discovered circRNAs should be validated by qPCR combined with either RNase R treatment or amplicon sequencing; and (6) combining the identification tools with annotation databases such as ExoRBase and circBase especially for exosomal circRNA research.

## The interplay between exosomal circRNAs and other ncRNAs: Functional crosstalk and involvement in cancer progression

Previous studies have shown that the expression patterns of some exosomal circRNAs are multifaceted and may demonstrate diverse functions among different cell types and tissues in various types of cancers [149, 150].

Exosomal circRNAs play contrasting roles depending on their cellular origin. Exosomal circRNAs derived from normal cells have been shown to inhibit cancer proliferation in recipient cells, while tumor-derived exosomal circRNAs could promote cell proliferation and malignancy progression. Abnormal expression of exosomal circRNAs is often associated with cancer initiation and progression [151]. For instance, circRNA_100284 has been implicated in the benign transformation of cells [152]. Additionally, circHIPK3, as reported by Zhou J. et al. (2021), regulates critical processes such as cell migration, differentiation, and proliferation, making it a pivotal player in cancer progression [153]. As discussed in previous sections, many circRNAs contribute to cancer progression and resistance through interactions with other RNAs, particularly miRNAs. This section will elaborate on additional examples of how circRNAs engage in crosstalk with miRNAs in various types of cancer.

### Hepatocellular carcinoma (HCC)

Exosomal circRNA_100284 was found to be involved in the malignant transformation of human hepatic cells through sponging miR-217, thus stimulating the downstream signaling cascade and upregulating the expression of EZH2 and cyclin-D1 in human hepatic cells. Overexpression of circRNA_100284 enhanced the formation, invasion, and migration of tumor colonies [152].

Liu et al. (2020) demonstrated that circMMP2 could be delivered to less invasive hepatocellular carcinoma (HCC) cells via exosomes derived from highly invasive HCC cells. CircMMP2 can upregulate the expression of its host gene matrix metallopeptidase 2 (MMP2) by sponging miR-136-5p, a metastasis-associated RNA that promotes HCC cell metastasis [154]. Similarly, circ_0051443 enhances cancer cell cycle arrest by interacting with miRNAs, as previously discussed [155]. CircUHRF1 was shown to enhance HCC proliferation, migration, invasion, and EMT in vitro and to promote tumor growth in vivo. Mechanistically, circUHRF1 was proposed to act as a sponge for miR-526b-5p, positively regulating the c-Myc protein. This was validated through experiments where transfection with a miR-526b-5p mimic led to a reduction in c-Myc levels, while using its inhibitor resulted in an increase in c-Myc levels. These findings confirm that c-Myc is a target of the circUHRF1/miR-526b-5p axis [64]. Exosomal circUHRF1 has also been involved in HCC treatment resistance through miR-449c-5p, as earlier discussed [101]. On the other hand, circ_0051443, transferred from normal cells to HCC cells via exosomes, reduces malignant characteristics by inducing cell death and halting the cell cycle. Meanwhile, exosomal circ-DB contributes to HCC progression by inhibiting miR-34a and activating USP7, a deubiquitination-related protein, which enhances tumor development and reduces DNA damage [156].

### Lung cancer

CircRNA-002178 was depicted to sponge miR-34 in malignant cells, thus enhancing programmed cell death ligand 1 (PDL1) expression [157]. Similarly, circSATB2 was identified to be differentially expressed in lung cancer tissues and serum exosomes. It promotes the development of non-small cell lung cancer (NSCLC) by upregulating fascin homolog 1 (FSCN1) and actin-bundling protein 1 via miR-326 regulation [158]. circFECR1 was also shown to promote tumor metastasis via the miR584-3p/ROCK1 pathway in SCLC cells. circFECR1 sequesters and inactivates the tumor suppressor miR584-3p, activating the ROCK1 gene and driving metastasis [159].

### Colorectal cancer (CRC)

According to Han et al. (2020), exosomal circRNAs have been shown to play critical roles in CRC by contributing to invasion, proliferation, metastasis, and apoptosis. Specifically, CRC-derived exosomal circPACRGL was found to stimulate CRC proliferation and metastasis by regulating the miR-142-3p/miR-506-3p-TGF-β1 axis [160]. Exosomal circ-133 from hypoxic cells, when transferred to normoxic cells, stimulated colorectal cancer metastasis by targeting the miR-133a/GEF-H1/RhoA axis [161]. Furthermore, exosomes from oxaliplatin-resistant cells delivered ciRS-122 to sensitive cells, thereby promoting glycolysis and drug resistance through miR-122 sponging and PKM2 upregulation [100].

In CRC, circ_0094343 acts as a sponge for miR-766-5p, which in turn targets and regulates TRIM67. There was a negative correlation between miR-766-5p and TRIM67 expression, while circ_0094343 was positively associated with TRIM67. This axis is crucial for cell proliferation, glycolysis, and chemosensitivity [85].

### Breast cancer (BC)

Mechanistically, circRHOT1 acted as a sponge for the tumor-suppressive miR-204-5p, leading to its downregulation and subsequent upregulation of protein arginine methyltransferase 5 (PRMT5), a protein known to drive tumor growth and metastasis. In a mouse model, circRHOT1 knockdown reduced tumor growth, decreased PRMT5 expression, and restored miR-204-5p levels. These findings underscore the significance of circRHOT1/miR-204-5p/PRMT5 axis in BC management [162].

### Ovarian cancer (OC)

In addition to the previously reported circPUM1 and CDR1as, which influence ovarian cancer (OC) progression and resistance via interactions with miR-615-5p, miR-6753-5p, and miR-1270/SCAI signaling pathways [163, 164], other circRNAs also interact with miRNAs impacting cancer progression. For example, circ_0001068, elevated in the serum exosomes of OC patients, acts as a ceRNA for miR-28-5p. Through exosomal transfer, it is delivered into T cells, where it increases PD1 expression, potentially influencing immune responses in OC [102]. Similarly, as noted in the previous sections, circWHSC1 was significantly elevated in OC tissues, especially in those with moderate to poor differentiation. It promotes cancer cells proliferation, migration, and invasion while inhibiting apoptosis. It was demonstrated to do so by acting as a sponge for miR-145 and miR-1182, leading to increased expression of downstream targets MUC1 and hTERT [165].

### Gastric cancer (GC)

CircNRIP1 acts as a tumor promotor in GC by absorbing miR-149-5p to influence AKT1 expression. Additionally, exosomes produced from gastric cancer cells activate PRDM16 and inhibit miR-133, by transferring ciRS-133 into preadipocytes and encouraging their differentiation into brown-like cells [166–168].

### Pancreatic cancer

In pancreatic ductal adenocarcinoma (PDAC) patients, exosomal circ-PDE8A was linked to both prognosis and progression. Through the MACC/MET/ERK or AKT pathways, Circ-PDE8A induces invasive growth and functions as a ceRNA for miR-338. Exosomal circPDE8A is released by tumor cells, which in turn promotes invasive advancements in pancreatic cancer cells in a manner dependent on the miR-338-MET transcriptional regulator MACC1-MET protooncogene receptor tyrosine pathway. This indicates that the transfer of circ-PDE8A facilitated by exosomes increases the invasiveness of tumors [169].

### Prostate cancer

CircRNAs in exosomes are essential for prostate cancer invasion and metastasis [170]. Circ_0044516 is upregulated in exosomes from prostate cancer patients, promoting cell growth and metastasis, potentially by sponging miR-29a-3p and controlling the downstream targets [171].

It is important to highlight that exosomal circRNAs extend their significance beyond cancer, playing a crucial role in neurological [172, 173] and cardiovascular diseases [174]. These molecules influence disease development and progression, serve as reliable biomarkers, and present promising opportunities for therapeutic intervention [175–179]. Their involvement in molecular regulation and intercellular communication underscores their broader impact on understanding and managing complex health conditions.

## Clinical implications and therapeutic potential of exosomal circRNAs

### Exosomal circRNAs as potential biomarkers for cancer diagnosis and prognosis

Exosomes are released into the bloodstream and other bodily fluids. Their stable structure and ability to transport molecules make them valuable indicators of tumor conditions [180]. Components within exosomes can serve as markers for detecting tumors and monitoring treatment effectiveness. Among these, exosomal circRNAs have gained attention for their unique properties and roles in biological processes [181]. Research shows that a significant proportion of circRNAs are enriched in blood exosomes. For example, analysis of human serum exosomes identified thousands of distinct circRNAs, far exceeding the concentration of linear RNAs, suggesting preferential enrichment [45]. One such case is ciRS-7, which is found in much higher concentrations in exosomes compared to its corresponding mRNA levels, indicating selective accumulation into exosomes [58]. These findings suggest that studying exosomal circRNAs provides a more accurate and detailed analysis than examining entire plasma or serum samples.

This section explores circRNAs’ clinical and therapeutic potential, focusing on their use as diagnostic biomarkers for early detection, prognosis, and therapeutic applications. Understanding their significance may pave the way for novel diagnostic and therapeutic strategies.

While studying circRNA in fluid exosomes is still in its early stages, numerous studies have demonstrated their potential as cancer biomarkers. Recent studies highlight the promising role of exosomal circRNAs as diagnostic markers in various types of cancer, such as breast, lung, colon, liver, and prostate cancer, with levels correlating to tumor stage, metastatic potential, and patient response [67, 110, 182].

A study by Kang et al. (2022) demonstrated that exosomal circRNA_0001492, circRNA_0001439, and circRNA_0000896 were elevated in the serum of lung adenocarcinoma patients compared to healthy individuals with the area under the curve (AUC) exceeding 0.75 for each of the three circRNAs individually. Their combination increased the AUC value to 0.805, indicating their potential as effective diagnostic biomarkers for lung adenocarcinoma [183]. In another study, exosomal circRNA_0047921, circRNA_0056285, and circRNA_0007761 significantly distinguished between NSCLC patients from healthy control. The combined panel of these circRNAs exhibited an AUC value of 0.919. Additionally, circRNA_0047921 effectively distinguished NSCLC cases from the chronic obstructive pulmonary disease group, while the combination of circRNA_0056285 and circRNA_0007761 were significantly different between NSCLC cases and the tuberculosis group. Moreover, circRNA_0056285 expression correlated with clinical stage and lymph node metastasis, highlighting its diagnostic relevance. Collectively, these circRNAs demonstrate significant potential for early-stage NSCLC diagnosis [184].

Li et al. (2019) observed that elevated levels of serum exosomal circFECR1 were associated with poor survival and clinical response to chemotherapy in small cell lung cancer (SCLC) patients. This suggests that circFECR1 could serve as a promising biomarker for tracking cancer progression. Notably, serum exosomal circFECR1 levels significantly decreased following first-line chemotherapy in patients with partial or complete remission, suggesting a dynamic correlation with chemotherapy response. These findings indicate that exosomal circFECR1 could be a valuable clinical marker for predicting treatment outcomes [159].

In CRC patients, circRNA_0003270 was significantly elevated in plasma exosomes with 75.64% sensitivity, 71.79% specificity and an AUC of 0.766. Additionally, the high level of circRNA_0003270 was positively correlated with lymph node metastasis and TNM stage. These results highlight the diagnostic value of circRNA_0003270 in CRC [185]. Exosomal circRNA_0004771, another exosomal circRNA, showed promise as a diagnostic biomarker for CRC. It had potential to differentiate between early stage and stage I/II CRC patients from healthy individuals with AUC of 0.88 and 0.59, respectively. It distinguished between patients with benign intestinal diseases and stage I/II CRC patients with AUC of 0.816. Additionally, exosomal circRNA_0004771 level was much lower in CRC patients after surgery, indicating that the elevated pre-operative level was possibly attributed to the presence of the tumor. Further, CRC cells treated with GW4869 (an exosomes secretion inhibitor) showed a down-regulation of exosomal hsa-circ-0004771 in the cultured media. These points highlight the significance of hsa-circ-0004771 as a biomarker for CRC, providing insights into its potential use in diagnosis and monitoring of the surgical intervention success [186].

The efficacy of exosomal circRNAs as biomarkers for BC has been also investigated. It was demonstrated that circRHOT1 was significantly elevated in exosomes derived from the serum of BC patients compared to healthy controls, demonstrating a diagnostic AUC of 0.83. The study also revealed that exosomes containing circRHOT1 promoted the growth, spread, and invasion of BC cells while suppressing apoptosis [162].

In HCC, circ_0051443 was significantly reduced in plasma exosomes compared to healthy controls. Transferred from normal cells to HCC cells via exosomes, circ_0051443 suppressed malignant behaviors by promoting cell death, and halting cell division. These effects suggest its potential as both a diagnostic and therapeutic target for HCC [155].

Exosomal circ_0028861 is another downregulated circRNA in HCC patients. It was established as a novel biomarker for HCC, as it distinguished between HCC patients and chronic hepatitis B (HBV) and cirrhosis with AUC of 0.79. It displayed sensitivity in detecting small, early stage, and alpha-fetoprotein-negative HCC tumors [182].

Elevated levels of plasma exosomal circCDR1as and circ_0015286 were observed in GC patients compared to healthy controls. Exosomal circCDR1as is associated with lymphatic metastasis and tumor progression. The diagnostic efficacy of circCDR1as was evaluated in GC patients, recording an AUC of 0.536, which increased to 0.786 when combined with serum carcinoembryonic antigen (CEA) and carbohydrate antigen 19–19 (CA19-9). These findings suggested that exosomal circCDR1as plays a role in GC development and propose it as a potential diagnostic and prognostic biomarker [187]. Regarding plasma exosomal circ_0015286, it had an AUC value of 0.778 to distinguish GC patients from healthy controls, which was higher than CEA (0.673) and CA19-9 (0.665). Combining these three markers further improved diagnostic accuracy, with an AUC of 0.843. The expression of circ_0015286 was closely linked to tumor size, TNM stage, and lymph node metastasis. Its levels decreased significantly following surgery. Patients with low circ_0015286 levels exhibited longer overall survival compared to those with higher levels. Hence, exosomal circ_0015286 could be a valuable noninvasive biomarker for the diagnosis and prognosis of GC [188].

As mentioned earlier, several studies have demonstrated a correlation between the levels of exosomal circRNAs in body fluids and the pathological features of tumor vascular invasion and TNM stage. Li et al. (2018) observed a significant elevation of circ-IARS in serum exosomes of patients with metastatic pancreatic cancer. This upregulation was associated with tumor vascular invasion, liver metastasis, and TNM stage. Survival analysis revealed a negative correlation between circ-IRAS expression and survival time, suggesting that circ-IRAS in serum exosomes could serve as a non-invasive biomarker for early diagnosis and prognosis of pancreatic cancer [189]. Another study depicted that the presence of exosomal circ-PDE8A in plasma was associated with tumor invasion and progression of PDAC patients, suggesting its potential use as a valuable marker for early diagnosis and prognosis of PDAC patients [169].

Further, serum exosomal circ_0026611 levels were significantly higher in esophageal squamous cell cancer (ESCC) patients with lymph node metastasis compared to those without. This circRNA could effectively distinguish between these two groups, with an AUC of 0.724. Patients with higher serum levels of exosomal circ_0026611 had poorer overall survival than those with low serum levels. These findings suggest that serum exosomal circ_0026611 is a valuable biomarker for ESCC prognosis [190].

In laryngeal squamous cell carcinoma (LSCC), Tian et al. demonstrated that circRASSF2 can promote the progression of LSCC. Clinical samples revealed significantly elevated levels of circRASSF2 in the serum exosomes of LSCC patients compared to healthy controls, highlighting its potential as a diagnostic marker for LSCC [191].

Circ_0001068 was found to be elevated in the serum exosomes of ovarian cancer (OC) patients. It demonstrated a high diagnostic potential with an AUC of 0.9697, indicating its potential effectiveness as a noninvasive biomarker for OC [102]. Further, it has been shown that circWHSC1 is also significantly elevated in OC tissues, particularly in those with moderate to poor differentiation. The findings suggest that circWHSC1 could serve as a potential biomarker for OC diagnosis and a therapeutic target, given its significant role in cancer progression and metastasis [165].

The diagnostic potential of exosomal circRNA in other body fluids was also studied. Urine, in particular, is widely used in clinical practice due to its easy collection and low detection costs. Studies have revealed elevated levels of circPRMT5 in urine exosomes of patients with bladder urothelial carcinoma compared to healthy ones. Moreover, circPRMT5 expression was correlated with tumor lymph node metastasis and progression, suggesting its potential as a diagnostic marker for this type of cancer [192]. The diagnostic potential of exosomal circRNAs in saliva for cancer remains uncertain. Nevertheless, recent research has suggested that circRNA-se*G*-*N*chiRNA in salivary exosomes could be a promising biomarker for early detection, treatment response evaluation, and relapse monitoring in ESCC [193]. To this end, it is anticipated that advancements in theory and technology will uncover further potential applications of exosomal circRNAs as tumor biomarkers. The previously mentioned studies are summarized in Table 2.

**Table 2:** Exosomal circRNAs as potential biomarkers for cancer diagnosis and prognosis

Exo-CircRNA	Diagnosis/ prognosis	Sample	Cancer type	Biomarker potential	Ref
CircRNA_0001492, 0001439, 0000896	Diagnosis	serum exosomal circRNAs (134 LUAD cases and 50 controls) and serum circRNAs (74 LUAD cases and 40 controls)	Lung adenocarcinoma	AUC > 0.75 for each individual circRNA. Their combination increased AUC to 0.805	[183]
CircRNA_0047921, 0056285, and 0007761	Early-stage diagnosis	30 NSCLC cases and 45 noncancerous controls	Non-small cell lung cancer	The combined panel of these circRNAs exhibited an AUC value of 0.919	[184]
CircFECR1	Diagnosis and prognosis (poor survival, positive LNM)	56 primary SCLC tissues and 50 NSCLC tissues	Small cell lung cancer	AUC not mentioned, FECR1 is a potential biomarker for tracking cancer progression	[159]
CircRNA_0003270	Diagnosis	Plasma (f 78 CRC patients and matched 78 healthy controls)	Colorectal cancer	75.64% sensitivity, 71.79 specificity and an AUC of 0.766	[185]
CircRNA_0004771	Diagnosis	Serum (135 CRC patients, 35 patients with benign intestinal diseases (BIDs) and 45 healthy controls)	Colorectal cancer	AUC of 0.88	[186]
CircRHOT1	Diagnosis	Serum (10 primary BC)	Breast cancer	AUC not mentioned	[162]
Circ_0051443	Diagnosis	60 patients with HCC and 60 healthy control subjects, together with 60 matched tumor and normal adjacent tissues from patients with HCC	Hepatocellular carcinoma	AUC not mentioned	[155]
Circ_0028861	Early-stage diagnosis	Serum (56 HCC patients, 47 patients with liver cirrhosis, and 57 chronically infected HBV patients)	Hepatocellular carcinoma	AUC of 0.79	[182]
CircCDR1as	Diagnosis	GC and adjacent normal tissues (n = 82), paired plasma (n = 65) and plasma exosome samples (n = 68) from GC patients and healthy controls	Gastric cancer	AUC of 0.536, increased to 0.786 when combined with CEA and CA19-9	[187]
Circ_0015286	Diagnosis and prognosis (overall survival)	Plasma (60 patients with newly diagnosed GC, 30 chronic gastritis patients and 30 healthy subjects)	Gastric cancer	AUC value of 0.778, AUC 0f 0.843 when combined with CEA and CA19-9	[188]
Circ-IARS	Early diagnosis and prognosis (TNM, postoperative survival time)	85 PDAC tissues, plasma exosomes	Metastatic pancreatic cancer	Negative correlation between circ-IRAS expression and survival time	[189]
CircPDE8A	Early diagnosis and prognosis (survival, LNM)	93 fresh frozen tissues and 20 peritumoral normal tissues and plasma	Pancreatic ductal adenocarcinoma	Positive correlation between plasma circ-PDE8A and tumor invasion and progression in PDAC patients	[169]
Circ_0026611	Prognosis (LNM)	Serum (69 samples from patients with ESCC)	Esophageal squamous cell cancer	AUC of 0.724 and poor overall survival	[190]
CircRASSF2	Diagnosis	85 pairs of tumor tissue and adjacent normal tissue from patients subjected to LSCC surgical resection	Laryngeal squamous cell carcinoma	Upregulated in serum exosomes of LSCC patients	[191]
Circ_0001068	Diagnosis	Twenty pairs of OC tissues and adjacent non-cancerous tissues & 95 OC patients who were not receiving any treatments and 53 matched healthy volunteers	Ovarian cancer	AUC of 0.9697	[102]
CircWHSC1	Diagnosis	79 epithelial ovarian carcinoma samples and 13 normal ovary samples	Ovarian cancer	Upregulated in tissues with moderate and poor differentiation	[165]
CircPRMT5	Diagnosis	Urine (119 patients with UCB)	Bladder/ Urothelial carcinoma	Positive correlation with tumor lymph node metastasis	[192]
CircRNA-se*G*-*N*chiRNA	Early detection	Saliva (10 patients & 10 healthy)	Esophageal squamous cell carcinoma	Positive correlation with poor differentiation, lymph node metastasis, and shorter overall survival	[193]

### Therapeutic applications and targeted delivery of exosomal circRNAs; immune modulation implication and tumor progression

Exosomes offer a promising approach for delivering targeted treatments to cancer cells. Exosome-associated miRNAs and several other molecules have been studied as ncRNA-based therapeutics [3, 13, 194]. Certain circRNAs possess tumor-suppressing properties, suggesting that exosomal delivery of these circRNAs could potentially inhibit tumor growth, progression, and drug resistance. If tackling tumor growth, progression or drug resistance could be via the tumor micro-environment or a specific tumor-related genes as the insulin-like growth factor or the glucagon-like peptide, others, or drug-transporting/metabolizing enzymes [195–200], respectively, are points worth further experimental and clinical research.

Conversely, some circRNAs promote malignant behaviors, making their inhibition therapeutically significant. Harnessing exosomes to deliver anticancer circRNAs or inhibiting oncogenic circRNAs presents a promising strategy for developing novel cancer treatments [201].

Exosomes isolated from CRC patients exhibit decreased levels of circEPB41L2. Notably, the transfer of circEPB41L2 via exosomes was found to inhibit tumor progression by modulating the PTEN/AKT signaling pathway. This suggests that increasing the levels of circEPB41L2 in CRC patients could potentially suppress tumor growth and metastasis, highlighting its significance as a therapeutic target for CRC [202]. It has been reported that circ_0094343 was significantly downregulated in CRC tissues, chemotherapy-resistant CRC tissues, and metastatic CRC tissues. Circ-0094343 inhibits the proliferation, colony formation, and glycolysis of CRC cells and enhances their chemosensitivity to various chemotherapeutic drugs. These findings may offer novel therapeutic avenues for the treatment of CRC [85]. Another study identified a novel circRNA, circPACRGL, derived from CRC exosomes. CircPACRGL promoted CRC cell proliferation, migration, invasion, and the differentiation of N1 to N2 neutrophils through the miR-142-3p/miR-506-3p-TGF-β1 axis as mentioned earlier. Therefore, targeting circPACRGL represents a promising therapeutic strategy for CRC [160, 203].

CirRHOBTB3 dysregulation was linked to tumorigenesis, metastasis, and chemoresistance in CRC. Researchers developed antisense oligonucleotides (ASOs) that may exert anti-tumor effects specifically by increasing the levels of cirRHOBTB3 within cells and preventing its release into exosomes. In a mouse model of liver metastasis, tail vein injection of a combination of ASO-cir and ASO-exo (a second generation ASO targeting circRNAs and exosome secretion elements, respectively) resulted in a significantly reduced body weight and less liver metastasis compared to the control group. These results suggest that targeting circRHOBTB3 production and release with ASOs could be a promising new treatment for CRC [204]. In contrast to circRHOBTB3, circSLC7A6 was found to stimulate the growth, spread, and survival of CRC cells. Martine, a natural alkaloid, was shown to reduce the growth of CRC tumors by preventing cancer-associated fibroblasts from releasing exosomes containing circSLC7A6. Additionally, circSLC7A6 was depicted to inhibit apoptosis while acting as a promoter of CRC cell invasion and proliferation [205].

Exosomal circZNF652 was elevated in the serum and cells of HCC patients. CircZNF652 could be transferred to HCC cells, and its silencing was shown to inhibit their proliferation, migration, invasion, and glycolysis. CircZNF652 was shown to act as a sponge for miR-29a-3p. Additionally, circZNF652 knockdown repressed tumor growth in vivo. Based on these data, it is proposed that circZNF652 exhibits significant potential as a therapeutic target for HCC [206]. The role of circ-002136 in HCC progression was identified by Yuan et al., who reported that it promotes HCC advancement by activating the miR-19a-3p/RAB1A pathway. Circ-002136 acts as a sponge for miR-19a-3p, leading to increased expression of RAB1A. RAB1A is a small GTPase involved in intracellular trafficking and has been implicated in cell proliferation and migration. By upregulating RAB1A, circ-002136 promotes HCC cell growth and invasion. These findings suggest that targeting circ-002136 or the miR-19a-3p/RAB1A pathway could potentially inhibit HCC progression, offering novel insights into HCC development and identifying potential therapeutic targets [207]. Furthermore, HCC cells co-cultured with macrophage exosomes targeting RBPJ exhibited increased tumor proliferation and body weight following subcutaneous injection into nude mice. RBPJ is a transcription factor involved in Notch signaling and known to contribute to cancer progression. These findings suggest that hsa_circ_0004658, carried by macrophage-derived exosomes, could serve as a promising therapeutic target for HCC. It functions as a ceRNA by sequestering miR-499b-5p, thereby de-repressing JAM3, which ultimately inhibits tumor growth and promotes apoptosis in HCC cells [208].

Macrophage-derived exosomes have been implicated in tumor progression, but their role in glioma remains unclear. A recent study demonstrated that circBTG2 is upregulated by RBP-J, which inhibits the proliferation and invasion of glioma cells, while circBTG2 knockdown promotes tumor growth in vivo. The effects of RBP-J macrophage-derived exosomes on glioma cells were reversed by circBTG2 knockdown. Exosomal circBTG2, secreted from RBP-J macrophages, inhibited tumor progression through the circBTG2/miR-25-3p/PTEN pathway, highlighting its potential therapeutic role in glioma [209]. High levels of circMMP1 have been found in exosomes released by glioma cells, where it promotes tumor growth by interacting with miR-433. CircMMP1 can sponge miR-433, thereby preventing its tumor suppressive effects. Hence, targeting this pathway might be a promising approach for treating glioma [150].

CircSTAU2 is significantly downregulated in GC. It inhibits GC cell proliferation, invasion, and migration, while its knockdown promotes tumor growth. Notably, circSTAU2 can be encapsulated within exosomes and delivered to cells where it acts as a sponge for miR-589, thereby inhibiting cancer progression. Furthermore, MBNL1 significantly influences the circulation and expression of circSTAU2, suggesting that targeting MBNL1 may also represent a therapeutic avenue. Therefore, exosome-delivered circSTAU2 could serve as a promising anti-tumor strategy by targeting miR-589 [210]. Additionally, researchers discovered that circRELL1 directly suppresses cell proliferation, invasion, and migration while promoting cell death. Mechanistically, circRELL1 binds to miR-637, indirectly increasing the levels of EPHB3 by regulating autophagy [211]. Additionally, Yang et al. observed that exosomes containing high levels of circUBE2Q2 can promote the spread of GC cells in mice. Reducing the levels of circUBE2Q2 in these mice slowed down tumor growth, and this effect was even stronger when combined with a drug that blocks STAT3, that is originally involved in promoting glycolysis and inhibiting autophagy [212]. Overall, these studies highlight the potential of circRNAs, such as circRELL1 and circUBE2Q2 as key regulators of GC progression. Their ability to be transmitted via exosomes suggests their potential as circulating biomarkers, as well as therapeutic targets. Exosomal circRanGAP1 demonstrated a pivotal role in the advancement of GC. The plasma exosomes derived from GC patients increased the migration and invasion of GC cells [213]. Additionally, circ-ITCH overexpression prevented GC cells from proliferating, migrating, invading, and undergoing the epithelial mesenchymal transition (EMT), while its knockdown had the reverse effect [214].

A recent study demonstrated that exosomes released by cancer-associated fibroblasts (CAFs) from OC can slow down the growth, spread, and transformation of OC cells. These CAFs directly transfer exosomes to OC cells, increasing the levels of circIFNGR2 within the cancer cells. The increase in circIFNGR2 blocks cell proliferation, metastasis, and endothelial-mesenchymal transition by activating miR-378, ultimately hindering the progression of OC [215]. Another circRNA, circPUM1, was shown to promote OC progression by increasing migration, invasion, and proliferation while inhibiting apoptosis in CAOV3 cells. Its overexpression facilitated cancer spread through cancer-derived exosomes compared to the control group. Mechanistically, circPUM1 acted by sponging miR-615-5p and miR-6753-5p, leading to increased expression of MMP2 and nuclear factor kappa B (NF-κB), which enhanced OC cell invasion, migration, and proliferation. Additionally, circPUM1 influenced the peritoneum and contributed to cancer metastasis via cancer-derived exosomes [163].

A study by Yao et al. (2021) revealed that exosomes released by bone marrow mesenchymal stem cells (BM-MSCs) can significantly slow down the growth, spread, and stemness of pancreatic cancer cells. Further analysis identified the involvement of circ_0030167 and its target miR-338-5p. It was shown that circ_0030167 primarily regulates miR-338-5p, which in turn inhibits the Wnt8/β-catenin signaling pathway, ultimately reducing the stemness of pancreatic cancer cells. This understanding could potentially lead to novel therapeutic approaches for treating pancreatic cancer [216]. Another study found that exosomes released by BM-MSCs can suppress the growth and spread of PDAC cells and their ability to evade the immune system. These exosomes increase the levels of circ_0006790 within PDAC cells. Blocking circ_0006790 significantly reduces the anti-tumor effects of these exosomes. Circ_0006790 promotes the movement of a protein called chromobox7 (CBX7) into the nucleus of PDAC cells. CBX7 then prevents S100A11 gene, leading to inhibition of growth, spread, and immune evasion of PDAC cells. Blocking CBX7 can reverse the anti-tumor effects of the exosomes. These findings suggest that targeting circ_0006790 or its associated pathways could be a potential therapeutic strategy for PDAC [217].

CircRNA_002178 is upregulated in lung adenocarcinoma tissues and cells, where it promotes PDL1 expression, leading to T-cell exhaustion [157]. Exosomal delivery of circRNA_002178 to CD + T cells also induces programmed cell death protein 1 (PD1) expression, highlighting its role in immune evasion. These findings suggest that circRNA_002178 acts as a crucial regulator of PDL1/PD1 expression in lung adenocarcinoma, offering a potential therapeutic target for this type of cancer. Furthermore, circRNA-002178 isolated from plasma exosomes of lung adenocarcinoma patients can be delivered to CD8 + T cells to stimulate PD1 expression, potentially impacting immune responses [157]. These findings highlight the critical roles of exosomal circRNAs in lung cancer biology and immune modulation.

To this end, it is well-demonstrated that exosomal circRNAs have a significant role in various cancer types’ development, including CRC, lung adenocarcinoma, NSCLC, SCLC, GC, glioma, pancreatic cancer, HCC, and OC. These studies highlight the therapeutic potential of exosomal circRNAs. By understanding the mechanisms through which circRNAs regulate cancer cell behavior and identifying those with tumor-suppressive properties, novel therapeutic strategies can be developed. Targeting exosomal circRNAs offers a promising approach for improving cancer treatment outcomes, particularly in cases with limited therapeutic options.

### Role of exosomal circRNAs in chemoresistance

Drug resistance is a major challenge in cancer therapy, as it can limit the effectiveness of treatments. Exosomal circRNAs have been implicated in conferring drug resistance in several cancer types. This could be achieved through various mechanisms: sponging miRNAs, altering gene expression, or modulating cellular signaling. CircRNAs can act as ceRNAs, binding to miRNAs. Further, circRNAs can directly interact with proteins or DNA to influence gene expression. In turn, these interactions might alter the expression of the genes involved in drug metabolism, efflux, or signaling pathways that contribute to drug resistance. Additionally, exosomal circRNAs can be transferred between cells, allowing them to influence the signaling pathways and cellular phenotypes of recipient cells. This can create a microenvironment that favors drug resistance [57, 218]. In the subsequent section, we will explore the significance and impact of exosomal circRNAs in mediating drug resistance across various cancer types.

CircUHRF1 is primarily released from HCC into the blood stream within exosomes and reduces the production of Interferon gamma (IFN-γ) and tumor necrosis factor- α (TNF-α) by natural killer (NK) cells. CircUHRF1 inhibits NK cell function by increasing the expression of TIM-3, a protein that helps cancer cells to evade the immune cells, by reducing the levels of miR-449c-5p. Additionally, this circRNA contributes to resistance to anti-PD1 immunotherapy in HCC patients. This suggests that circUHRF1 plays a role in promoting cancer progression and resistance to immunotherapy. Therefore, targeting circUHRF1 could be a promising new approach for treating HCC [101].

In another study, it was found that circSORE is transported by exosomes to spread sorafenib resistance among HCC cells. CircSORE was reported to bind YBX1 in the cytoplasm and prevent its degradation leading to increased tumor growth and metastasis. Therefore, silencing circSORE by siRNA injection could substantially overcome sorafenib resistance [99].

An immunosuppressive microenvironment and resistance to anti-PD1 immunotherapy were promoted by exosomal circTMEM181 secreted by HCC cells. CircTMEM181 sponges miR-488-3p, upregulating CD39 expression on macrophages leading to anti-PD1 resistance. So, targeting circTMEM181/CD39 axis represents a promising therapeutic strategy to overcome immune evasion and enhance the efficacy of anti-PD1 immunotherapy in HCC patients [219]. The potential therapeutic benefits of exosomal circRNA_G004213 in HCC were explored by Qin et al. (2021). They identified that circRNA_G004213 enhances the sensitivity of HCC cells to cisplatin treatment through the miR-513b-5p/PRPF39 pathway. The knockdown of PRPF39 expression using siRNA resulted in a significant increase in cisplatin resistance. Based on this finding, HCC patients could be sensitized to cisplatin treatment by targeting circRNA_G004213 [220].

In CRC, circ_0005963 was positively correlated with chemoresistance. The study demonstrated that exosomes derived from oxaliplatin-resistant CRC cells transferred circ_0005963 to sensitive cells, promoting glycolysis and drug resistance through miR-122 sponging and PKM2 upregulation. PKM2 overexpression leads to increased glycolysis, helping cancer cells to survive and proliferate despite the presence of chemotherapy [100]. This highlights the impact of circ_0005963/miR-122/PKM2 pathway in oxaliplatin drug resistance and opens the door to the development of potential effective strategies [66]. Another study by Pan et al. (2022) highlighted the role of circRNA in oxaliplatin resistance in CRC. They were able to show that circRNA-ATG4B was elevated in exosomes secreted by oxaliplatin-resistant CRC cells. They also reported that circRNA-ATG4B promoted autophagy and enhanced chemoresistance by encoding circRNA-ATG4B-222aa protein [75]. Further, exosomal circ_0000338 enhances 5- fluorouracil (5-FU) resistance by negatively regulating miR-217 and miR-485-3p. By downregulating these miRNAs, circ_0000338 was able to inhibit apoptosis and enhance 5-FU resistance in CRC cells [221]. Furthermore, exosomes from oxaliplatin-resistant CRC cells delivered ciRS-122 to sensitive cells, thereby promoting glycolysis and drug resistance through miR-122 sponging and PKM2 upregulation [100]. It has also been demonstrated that circ_0094343 improved the chemosensitivity of HCT116 cells to several chemotherapeutic drugs, including 5-fluorouracil (5-FU), oxaliplatin (L-OHP), and doxorubicin (Dox). This suggests that circ_0094343 could be a valuable target for enhancing the effectiveness of chemotherapy in CRC patients [85]. It has also been shown that following radiotherapy, an increase in exosomal circ_0067835 was observed in the serum of CRC patients. It was shown to sponge miR-296-5p, which in turn regulates the expression of insulin-like growth factor 1 receptor (IGF-1R). This interaction plays a crucial role in CRC cell proliferation, cell cycle progression, and apoptosis. Additionally, circ_0067835 suppression was depicted to further improve cell apoptosis and radiosensitivity in vitro and decrease cell proliferation and cell cycle progression [222].

In glioma patients undergoing radiotherapy, exosomal circATP8B4 was shown to sequester miR-766 and contribute to radiotherapy resistance. MiR-766 is known to play a role in regulating the sensitivity of cells to radiotherapy. This suggests that targeting circATP8B4 or its associated pathway could potentially improve the effectiveness of radiotherapy for glioma patients [223]. Exosomes released by temozolomide (TMZ)-resistant glioma cells were reported to be enriched with circ_0042003, which is positively regulated by heparinase enzyme, which is overexpressed in TMZ resistant cells. This data suggests that circ_0042003 is involved in TMZ resistance [224]. Circ_0072083 is another circRNA reported to be overexpressed in TMZ resistant glioma cells. Its downregulation enhances the sensitivity of glioma cells to TMZ by inhibiting tumor growth and promoting apoptosis. Circ_0072083 enhances this resistance by regulating NANOG expression through miR-1252-5p-mediated DNA methylation. Targeting circ_0072083 or its associated pathways represents a promising therapeutic avenue for overcoming TMZ resistance in glioma [225]. A third cirRNA, circHIPK3, was found to be elevated in TMZ resistance in glioma patients. Elevated CircHIPK3 levels directly target miR-421 reducing its expression in glioma patients. This effect can be reversed by ZIC5 protein upregulation, which counteracts the effects of miR-421 on cell growth and TMZ resistance. By reducing circHIPK3, researchers were able to slow down glioma cells growth. This was likely due to the increased levels of miR-421 and the subsequent reduction in ZIC5 [226].

CircZNF91 is overexpressed in gemcitabine (GME)-resistant pancreatic cancer cells. Exosomal circZNF91 can be transferred to neighboring cells, where it acts as a ceRNA for miR-23b-3p and depresses SIRT1, leading to increased glycolysis. This metabolic reprogramming contributes to GME chemoresistance in pancreatic cancer cells, which can be reversed by downregulating circZNF91 or upregulating miR-23b-3p. These findings highlight the pivotal role of exosomal circZNF91 in mediating GME chemoresistance in pancreatic cancer and suggest potential therapeutic strategies targeting this pathway [227].

In cisplatin-resistant NSCLC cells, circ_0076305 is overexpressed. Circ_0076305 acts as a sponge for miR-186-5p and consequently upregulates ABCC1, an efflux transporter that confers chemoresistance. These results suggest that targeting circ_0076305 could represent a novel therapeutic approach to overcome cisplatin resistance in NSCLC. By downregulating exosomal circ_0076305, it is possible to enhance cisplatin sensitivity by inhibiting NSCLC cell proliferation, migration, invasion, and promoting apoptosis [228]. Another study revealed a relation between circRNAs and ABCC1 in NSCLC patients. CircPIP5K1A and ABCC1 were elevated, while miR-101 was decreased, in NSCLC tissues, serum, and cells. CircPIP5K1A acts as a sponge for miR-101 and consequently upregulates ABCC1, a known drug-resistance gene [229]. Further, it was demonstrated that exosomes released by NSCLC cells that are resistant to epidermal growth factor receptor (EGFR) inhibitors (EGFR-TKIs), contain high levels of circ_102481. Increasing the level of circ_102481 in cells that are sensitive to EGFR-TKIs can promote their growth and inhibit cell death. Circ_102481 works by blocking miR-30a-5p, which regulates ROR1 protein expression, affecting cancer cell growth and survival. Therefore, measuring circ_102481 levels in exosomes could be a valuable tool for diagnosing EGFR-TKIs resistance in NSCLC and for developing new targeted therapies [91]. A research group discovered that exosomal circ_0002130 acts as a sponge for miR-498, leading to the regulation of glucose transporter 1 (GLUT1), hexokinase 2 (HK2), and lactate dehydrogenase A (LDHA) metabolic enzymes that promote glycolysis and confer resistance to osimertinib, an EGFR inhibitor. This suggests that exosomal circ_0002130 may also play a crucial role in mediating drug resistance mechanisms in NSCLC [230].

It has also been demonstrated that the exosomal transfer of circUBE2D2 contributes to tamoxifen resistance in BC by binding to miR-200a-3p and influencing cell viability, metastasis, and estrogen receptor alpha (ERα) levels, both in vitro and in vivo. This suggests potential new strategies for improving the effectiveness of tamoxifen treatment in BC [231].

It was found that circBACH1 levels were increased in exosomes derived from paclitaxel (PTX) treated BC cells and tissue. Exosomes containing circBACH1 promoted PTX resistance, stemness, and migration of BC cells through the regulation of miR-217 and GTPase-activating protein binding protein 2 (G3BP2). G3BP2 is known to induce tumor growth and survival. Targeting this pathway could be a potential therapeutic strategy for BC [232]. Another study highlighted the role of circ-CREIT in doxorubicin-resistant TNBC. Results showed that circ-CREIT levels were low in TNBC cells and linked to poor prognosis. Overexpression of circ-CREIT increased doxorubicin sensitivity in TNBC cells by promoting PKR degradation. PKR is a protein involved in stress granule formation and cancer cell survival. By targeting circ-CREIT and PKR protein, doxorubicin resistance could be avoided in TNBC cells [233].

Exosomal circPIP5K1A was overexpressed in cisplatin-resistant OC tissues and cells. Silencing circPIP5K1A suppressed proliferation, migration, and invasion, and increased sensitivity to cisplatin in cisplatin-resistant OC cells. This effect of circPIP5K1A was mediated through sponging miR-942-5p leading to upregulation of nuclear factor IB (NFIB) expression. Hence, targeting circPIP5K1A, either directly or through its interaction with miR-942-5p and NFIB, may represent an effective novel approach to improve treatment outcomes in OC patients [234]. Several studies have demonstrated that circRNAs are differentially expressed in various OC tissues, highlighting their significant role in OC progression. In cisplatin-resistant patient tissues and cell lines, circRNA CDR1as was found to be downregulated. Furthermore, serum exosomes from cisplatin-resistant patients also showed reduced CDR1as levels. Overexpression of CDR1as inhibited cell proliferation and promoted cisplatin-induced apoptosis in OC cells by regulating the miR-1270/SCAI signaling pathway [164].

CircXIAP was elevated, while miR-1182 was downregulated in docetaxel (DTX)-resistant prostate cancer tissues and cell lines. CircXIAP directly targets miR-1182, and its knockdown effects were reversed by downregulating miR-1182. Notably, circXIAP depletion inhibited tumor growth and increased DTX sensitivity in vivo, suggesting that targeting circXIAP may be a promising therapeutic strategy for enhancing DTX efficiency in prostate cancer patients [235].

To this end, it is quite evident that understanding the underlying mechanisms of exosomal circRNAs in mediating drug resistance is essential for developing effective strategies to overcome this critical clinical challenge. Additionally, some exosomal circRNAs can contribute to the creation of immunosuppressive microenvironments, thereby hindering the efficacy of immune-based therapies. Targeting these circRNAs or their associated pathways represents a promising approach for developing novel therapeutic strategies to enhance cancer treatment outcomes. The role of exosomal circRNAs in regulating drug resistance is summarized in Table 3.

**Table 3:** Role of exosomal circRNAs in drug resistance

CircRNA	CircRNA role	Samples used	Target	Cancer type	Ref
CircUHRF1	Promotes resistance to (Opdivo) anti-PD1 immunotherapy	6 HCC cell lines and serum samples	Reduces the production of IFN-γ and TNF-α by NK cells and downregulates miR-449c-5p	HCC	[101]
CircSORE	Promotes resistance to Sorafenib	In vivo & in vitro	Binds YBX1 in the cytoplasm and prevents its degradation	HCC	[99]
CircTMEM181	Promotes resistance to anti-PD1 immunotherapy	HCC cell lines and tissue samples	Sponges miR-488-3p, upregulating CD39 expression on macrophages	HCC	[219]
CircRNA-G004213	Enhances treatment sensitivity to Cisplatin	Blood samples	MiR-513b-5p/ PRPF39 pathway	HCC	[220]
Circ_0005963	Promotes resistance to adjuvant fluoro-pyrimidine and oxaliplatin chemotherapy (FOLFOX)	Tissue samples	Promotes glycolysis through miR-122 sponging and PKM2 upregulation	Metastatic CRC	[66, 100]
CircRNA_ATG4B	Promotes resistance to Oxaliplatin	Surgical tissue specimens & CRC cell lines	Promotes autophagy by encoding circRNA-ATG4B-222aa	CRC	[75]
Circ_0000338	Promotes resistance to 5-fluorouracil (5-FU)	Blood & tumor tissues	Negatively regulates miR-217 and miR-485-3p	CRC	[221]
Circ_0067835	Promotes radiotherapy resistance	39 CRC tissues and adjacent normal tissues (ANT) & 19 blood samples	Sponges miR-296-5p, regulating the expression of insulin-like growth factor 1 receptor	CRC	[222]
CircATP8B4	Promotes radiotherapy resistance	Cell lines	Sequester miR-766	Glioma	[223]
Circ_0042003	Promotes resistance to Temozolomide (TMZ)	Cell lines & tissue samples	Positively regulated by the heparinase enzyme of the resistant cells	Advanced glioma	[224]
Circ_0072083	Promotes resistance to TMZ	Blood & tissue samples	Regulates NANOG expression through miR-1252-5p-mediated DNA methylation	Glioma	[225]
CircHIPK3	Elevated in TMZ resistance	Blood samples	Directly targets miR-421 reducing its expression	Glioma	[226]
CircZNF91	Elevated in resistance to Gemcitabine (GME)	Blood & tissue samples	Acts as a competing endogenous RNA (ceRNA) for miR-23b-3p, depresses SIRT1, increasing glycolysis	Pancreatic cancer	[227]
Circ_0076305	Promotes resistance to Cisplatin	Cisplatin-resistant NSCLC tissues and cells	Acts as a sponge for miR-186-5p and consequently upregulating ABCC1	NSCLC	[228]
CircPIP5K1A	Promotes resistance to Cisplatin	Tissues and cells	Acts as a sponge for miR-101 and consequently upregulating ABCC1	NSCLC	[229]
Circ_102481	Promotes resistance to EGFR inhibitor (EGFR-TKIs); (gefitinib or erlotinib)	Blood samples & cell lines	Blocks miR-30a-5p, the ROR1 protein expression	NSCLC	[91]
Circ_0002130	Promotes resistance to Osimertinib (EGFR inhibitor)	Tissue samples & two lung adenocarcinoma cell lines	Acts as a sponge for miR-498 leading to the regulation of GLUT1, HK2, and LDHA enzymes	NSCLC	[230]
CircUBE2D2	Promotes resistance to Tamoxifen (TAM)-based neo-adjuvant chemotherapy	3 cell lines and tissue samples	Binds to miR-200a-3p influencing cell viability, metastasis, and estrogen receptor alpha (Erα) levels	BC	[231]
CircBACH1	Promotes resistance to Paclitaxel	Blood & tissue samples and cell lines	Regulates of miR-217 and Ras GTPase-activating protein binding protein 2 (G3BP2)	BC	[232]
Circ-CREIT	Increased doxorubicin sensitivity	Blood & tissue samples and cell lines	Promotes PKR degradation, a protein involved in stress granule formation and cancer cell survival	TNBC	[233]
CircPIP5K1A	Promotes resistance to Cisplatin	Blood & tissue samples and cell lines	Sponges miR-942-5p leading to upregulation of NFIB expression	OC	[234]
CDR1as	Decreased in resistance to Cisplatin	Blood & tissue samples and 3 cell lines	Regulates miR-1270/SCAI pathway	OC	[164]
CircXIAP	Promotes resistance to Docetaxel (DTX)	5 cell lines and tissue samples	Directly targets miR-1182	PC	[235]

[PC; prostate cancer, OC; ovarian cancer, TNBC; triple negative breast cancer, BC; breast cancer, NSCLC; non-small cell lung cancer, CRC; colorectal cancer, HCC; hepatocellular carcinoma.]

While the clinical promise of exosomal circRNAs is evident across diagnosis, prognosis, and therapy resistance, it is important to critically appraise the evidence. The majority of studies to date are exploratory, often retrospective in design, with small sample sizes that limit generalizability. Robust, prospective trials validating the diagnostic accuracy of exosomal circRNAs against established biomarkers (e.g., CEA, CA125) are still lacking. Similarly, therapeutic applications—whether through engineered delivery of tumor-suppressive circRNAs or inhibition of oncogenic species—remain at the conceptual or preclinical stage. Distinguishing between mechanistic insights from model systems and clinically validated findings is crucial for advancing the field toward translation.

## Clinical trials on exosomal circRNA

When searching ClinicalTrials.gov in September 2024, for the term "Exosomes" in the intervention category, specifically for the disease "cancer," and including "RNA" as an additional search term, 24 results were the output. However, a search for "cancer" and "exosomal circRNA" returned no results. Several obstacles face the application of exosomal circRNA in clinical trials. The lack of clinical trials reflects the fact that the field is moving through the traditional pipeline of scientific discovery, which moves from basic research to technical development, then translational validation, and finally clinical application. We are currently in the transition between the steps earlier to the clinical trial.

## Several factors are participating in the delay of this step, including:

### Focusing on basic research

Despite being identified decades ago, circular RNAs were mainly disregarded because of technological constraints and the belief that they were splicing-related non-functional byproducts [236]. The great majority of ongoing research is still in the fundamental science stage and focuses on identifying and classifying circRNAs in different disorders as well as comprehending their biogenesis and roles. It takes a long time to go from these fundamental discoveries to interventional clinical studies.

### Technical and analytical challenges

It is still difficult to separate pure populations of exosomes from blood and blood products (serum, plasma, and platelets) and bodily fluids (saliva, feces, and urine). Co-isolation of contaminants like lipoproteins or protein aggregates is common and can hinder downstream analysis, increasing the need for rigorous characterization. Furthermore, circRNAs frequently exhibit low levels of expression. Standard RNA-sequence methods and PCR assays are biased towards linear RNAs. More recently, next-generation sequencing approaches were introduced. Combining specific enrichment/detection/amplification approaches compatible with long-read sequencing and bespoke bioinformatics pipelines offers the most robust approach to investigate circRNA variants [237, 238]. This makes accurately quantifying specific circRNAs require specialized methods, which is not yet routine in clinical labs.

### Absence of universally standardized detection methods

For a biomarker to be used in a clinical trial, its measurement must be reproducible and standardized across multiple laboratories. There are no universally accepted standard operating procedures (SOPs) for this. Different RNA extraction kits, PCR platforms, and data normalization methods yield different results. Without consensus on the " standardized, validated protocol" to do it, data from different studies cannot be compared, making it impossible to define a clear-cut diagnostic or prognostic threshold for a trial.

### Complex biology and mechanistic uncertainty of exsosomal circRNAs

Advancing circRNA-based therapeutics requires a clear understanding of the mechanism of action. However, many exosomal circRNAs are multifunctional; a single molecule can bind RNA-binding proteins, act as a miRNA sponge, and, in some cases, be translated into peptides. Functions are also highly context-dependent; individual circRNAs may suppress tumor growth in one cancer type yet exhibit oncogenic activity in another. Initiating trials to inhibit or augment a circRNA without robust, context-specific mechanistic data in the intended patient population is therefore risky [239].

Moreover, although exosomes provide a natural delivery vehicle, substantial engineering is still required to achieve reliable loading of a defined therapeutic circRNA, selective tissue targeting, and minimization of off-target effects [240]. These uncertainties underscore the need for rigorous functional validation, disease- and subtype-specific characterization, and careful patient stratification before clinical translation.

## Technical challenges and future directions in harnessing exosomal circRNAs for clinical applications

A common limitation in current research is the failure to distinguish between exploratory mechanistic studies and clinically validated evidence. Without this distinction, the field risks overestimating the development of exosomal circRNA applications. Future research should follow a tiered approach, moving from in vitro discovery to in vivo functional validation and, eventually, to large-scale clinical trials. This strategy will help establish the robustness, reproducibility, and clinical utility of exosomal circRNAs.

The application of exosomal circRNAs as clinical biomarkers faces several technical challenges (Table 4). These stem from the heterogeneity of exosome populations, variability in their molecular cargo, lack of cell-of-origin–specific markers, and the fragility of vesicles during processing. Furthermore, current isolation methods are labor-intensive, expensive, and not well-suited for large-scale isolation or routine diagnostic use. Several emerging technologies have been developed that can address these limitations [113, 241, 242].

**Table 4:** Key issues hindering clinical translation of exosomal circRNAs

Knowledge gap category	Specific questions
Biogenesis and selective sorting	• Which circRNAs are loaded into exosomes? • Is the loading mechanism an active, regulated process for cell-to-cell communication, or a passive byproduct of cellular waste disposal • How does the loading mechanism differ in cancer cells compared to normal ones?
Functional mechanism	• What is their primary mechanism of action in vivo? • Do they mainly act through miRNA sponging, protein scaffolding, translation, or a mix of these functions?? • What are the exact mechanisms through which exosomal circRNAs alter the transcriptome and phenotype of recipient cells? • What influences the functional outcome in recipient cells (either oncogenic or tumor suppressor)?
Heterogeneity and specificity	• Can we define exosomal circRNA signatures that are specific to cancer type/ subtype? • How does the exosomal circRNA cargo change dynamically in response to therapy, hypoxia, or other tumor microenvironment stresses? • What is the contribution of non-tumor cells (e.g., cancer-associated fibroblasts, immune cells) to the total exosomal circRNA pool?
Technical and analytical	• What are the optimal, standardized protocols for isolation, detection, and quantification to ensure reproducibility across clinics? • How can tumor-derived exosomal circRNAs be distinguished from those released by normal tissues? • How do the baseline levels of particular exosomal circRNAs change with age, sex, or comorbidities in healthy individuals?

Droplet digital PCR (ddPCR), for example, offers single-molecule sensitivity and precise quantification of rare variants, making it an attractive technique for detecting circRNAs harboring cancer-relevant mutations [243]. Its relatively high per-sample cost, specialized instrumentation requirements, and limited throughput reduce its utility for large-scale screening. Currently, ddPCR is more suitable as a confirmatory tool rather than a primary diagnostic platform.

Microfluidic approaches, such as the immunomagnetic exosome RNA (iMER) system, successfully integrate exosome enrichment, RNA isolation, and RT-PCR on a single chip. These platforms minimize sample loss and processing time, but their use is still largely limited to proof-of-concept studies. Challenges to widespread adoption include the complexity of fabrication, the need for specialized instrumentation, and the absence of standardized workflows across different laboratories. While microfluidics presents a promising direction for automation and miniaturization, significant obstacles remain in terms of scalability and regulatory approval [244].

Ion-exchange and surface acoustic wave (SAW)-based microfluidic devices offer reductions in processing time and chemical interference, but their clinical applicability is constrained by limited throughput and the absence of validated pipelines for reproducible circRNA detection. Similarly, localized surface plasmon resonance (LSPR)-based assays achieve remarkable sensitivity for nucleic acid detection, yet they require sophisticated nanofabrication and optical detection systems not currently feasible in most clinical laboratories [245] [246] [247]. These platforms are powerful for research but need improvement in cost, robustness, and portability before widespread clinical implementation.

High-throughput sequencing remains the most comprehensive strategy for circRNA discovery and characterization, and dedicated circRNA-seq pipelines offered by commercial providers are making this technology increasingly accessible. Sequencing-based approaches continue to face challenges such as bioinformatic interpretation, inconsistencies in databases, and the necessity for standardized analysis pipelines to minimize false positives. Additionally, cost remains an issue, although prices are decreasing due to advances in sequencing technology.

The approaches mentioned illustrate both the advancements and limitations of current efforts to implement exosomal circRNAs in clinical applications. While each emerging technology presents unique benefits, none have fully satisfied the essential requirements for widespread clinical use: scalability, cost-effectiveness, reproducibility, and regulatory compliance. In the near term, ddPCR and sequencing are the most likely candidates for translational use, particularly in confirmatory diagnostics and exploratory biomarker panels. Microfluidic and nanoplasmonic systems, though promising, require further optimization and standardization before they can be integrated into precision oncology pipelines [248, 249]. A balanced perspective is therefore essential: technological innovation must be evaluated not only by analytical performance but also by feasibility, cost, and sustainability if exosomal circRNAs are to transition from experimental tools to clinically actionable biomarkers.

## Conclusion

Exosomal circRNAs have emerged as crucial molecules in the advancement of cancer diagnostics and therapeutics. Their unique circular structure and encapsulation within exosomes make them highly stable, resistant to degradation, and capable of facilitating intercellular communication. These properties place them as promising non-invasive biomarkers for cancers.

Beyond the technical and translational priorities discussed above, our review offers a conceptual perspective that sets it apart from previous work. We suggest that exosomal circRNAs should not be seen simply as extracellular byproducts of intracellular circRNA activity, but rather as a functionally distinct subclass in their own right. Encapsulation within exosomes gives these molecules unique advantages to act as mobile regulators, capable of carrying regulatory signals across cell boundaries and controlling gene expression in recipient cells. This way of thinking shifts circRNAs from being viewed as static, cell-confined molecules to being recognized as dynamic mediators of intercellular communication, connecting them directly to processes such as tumor-microenvironment crosstalk, immune modulation, and therapy resistance [250–253]. By bringing together insights on their biogenesis, mechanisms of action, detection technologies, and clinical applications, we propose that exosomal circRNAs are more than just another piece of the non-coding RNA puzzle. Instead, they represent a new paradigm with significant potential for translation into practice as innovative, actionable targets in precision oncology, opening new opportunities for personalized medicine.

Despite these promising findings, significant challenges remain in isolating and profiling exosomal circRNAs. Critical aspects such as cost considerations, scalability, and regulatory challenges remain important areas for further investigation. Furthermore, understanding the complex interactions between exosomal circRNAs and other cellular molecules, including other non-coding RNAs, is crucial for unraveling their complex regulatory networks and impact on cancer progression and treatment responses. In this context, it is also important to point out the rapid progress in small RNA therapeutics. The development of exosomal circRNAs as therapeutic agents aligns with the broader advancements in small RNA therapeutics, including siRNAs and miRNA-based drugs, which aim to modulate gene expression for cancer treatment. The stability and targeted delivery afforded by exosomes could potentially overcome some of the hurdles faced by other RNA-based therapies. To ensure clinical feasibility, further research into the stability, delivery efficiency, and off-target effects of exosomal circRNAs as RNA therapeutics is required. Although the potential involvement of exosomal circRNAs in non-cancerous disorders is not discussed here, they constitute a valuable area for future investigation. Emerging technologies, including advanced sequencing techniques and microfluidics, are expected to overcome major barriers hindering the wide translational applications. Advancements in this field highlight how exosomal circRNAs could transform precision medicine.

## Data Availability

No datasets were generated or analysed during the current study.

## Abbreviations

5-FU: 5-Fluorouracil
ASOs: Antisense oligonucleotides
AUC: Area under the curve
BM-MSCs: Bone marrow mesenchymal stem cells
BC: Breast cancer
CA19-9: Carbohydrate antigen 19–9
CAD: Coronary artery disease
CAFs: Cancer-associated fibroblasts
CBX7: Chromobox7
CEA: Carcinoembryonic antigen
ceRNAs: Competing endogenous RNAs
circRNAs: Circular RNAs
CiRNA: Intron circRNA
CiRS-7: Circular RNA sponge for miR-7
CMVECs: Cardiac microvascular endothelial cells
CRC: Colorectal cancer
CSF: Cerebrospinal fluid
CVC: Current-voltage characteristics
CVDs: Cardiovascular diseases
DLD: Deterministic lateral displacement
DTX: Docetaxel
EciRNA: Exon circRNA
EGFR: Epidermal growth factor receptor
EIciRNA: Exon–Intron circRNA
EMT: Epithelial mesenchymal transition
ERα: Estrogen receptor alpha
eRNAs: Enhancer RNAs
ESCC: Esophageal squamous cell cancer
EVs: Extracellular vesicles
exRNA: Exosomal RNA
G3BP2: GTPase-activating protein binding protein 2
GC: Gastric cancer
GME: Gemcitabine
GLUT1: Glucose transporter 1
HCC: Hepatocellular carcinoma
HK2: Hexokinase 2
IFN-γ: Interferon gamma
IGF-1R: Insulin-like growth factor 1 receptor
iMER: Immunomagnetic exosome RNA
IRES: Internal ribosomal entry site
LDHA: Lactate dehydrogenase A
lncRNAs: Long non-coding RNAs
LSCC: Laryngeal squamous cell carcinoma
LSPR: Localized surface plasmon resonance
miR: MicroRNA
miRNAs: MicroRNAs
MMP2: Matrix metallopeptidase 2
MRE: MiRNA response element
MVBs: Multivesicular bodies
ncRNAs: Non-coding RNAs
NFIB: Nuclear factor IB
NF-κB: Nuclear factor kappa B
NGS: Next-generation sequencing
NK: Natural killer
NSCLC: Non-small cell lung cancer
PTX: Paclitaxel
PCR: Polymerase chain reaction
PDAC: Pancreatic ductal adenocarcinoma
PDL1: Programmed cell death ligand 1
PD1: Programmed cell death protein 1
piRNA: Piwi-interacting RNAs
PKM2: Pyruvate kinase M2
PRMT5: Protein arginine methyltransferase 5
RBPs: RNA binding proteins
RT-PCR: Reverse transcription polymerase chain reaction
SAWs: Surface acoustic waves
siRNA: Small interfering RNAs
snoRNA: Small nucleolar RNA
snRNA: Small nuclear RNA
TMEM: Transmembrane protein
TMZ: Temozolomide
TNBC: Triple negative breast cancer
TNF-α: Tumor necrosis factor alpha
UTRs: Untranslated regions
VEGF: Vascular endothelial growth factor
